# Universal glass-forming behavior of *in vitro* and *living* cytoplasm

**DOI:** 10.1038/s41598-017-14883-y

**Published:** 2017-11-09

**Authors:** Kenji Nishizawa, Kei Fujiwara, Masahiro Ikenaga, Nobushige Nakajo, Miho Yanagisawa, Daisuke Mizuno

**Affiliations:** 1 Department of Physics, Graduate School of Sciences, Kyushu University, Fukuoka, 819-0395 Japan; 2 Department of Biosciences & Informatics, Keio University, Yokohama, 223-8522 Japan; 3 Department of Biology, Graduate School of Sciences, Kyushu University, Fukuoka, 819-0395 Japan; 4 Department of Applied Physics, Tokyo University of Agriculture and Technology, Tokyo, 184-8588 Japan

## Abstract

Physiological processes in cells are performed efficiently without getting jammed although cytoplasm is highly crowded with various macromolecules. Elucidating the physical machinery is challenging because the interior of a cell is so complex and driven far from equilibrium by metabolic activities. Here, we studied the mechanics of *in vitro* and *living* cytoplasm using the particle-tracking and manipulation technique. The molecular crowding effect on cytoplasmic mechanics was selectively studied by preparing simple *in vitro* models of cytoplasm from which both the metabolism and cytoskeletons were removed. We obtained direct evidence of the cytoplasmic glass transition; a dramatic increase in viscosity upon crowding quantitatively conformed to the super-Arrhenius formula, which is typical for fragile colloidal suspensions close to jamming. Furthermore, the glass-forming behaviors were found to be universally conserved in all the cytoplasm samples that originated from different species and developmental stages; they showed the same tendency for diverging at the macromolecule concentrations relevant for living cells. Notably, such fragile behavior disappeared in metabolically active living cells whose viscosity showed a genuine Arrhenius increase as in typical strong glass formers. Being actively driven by metabolism, the *living* cytoplasm forms glass that is fundamentally different from that of its *non-living* counterpart.

## Introduction

Elucidating the mechanics of the cytoplasm is essential for understanding cell behaviors because cytoplasm governs the dynamics of core functional molecules essential for living systems^1–3^. Active biomolecules such as enzymes and reactants must be transported via the cytoplasm and supplied at appropriate occasions and locations in cells. Physical agitation in the cytoplasm leads to jiggling of intramolecular conformations of proteins that are crucial to initiate biochemical reactions and facilitate subsequent signaling cascades. Cells that do not undergo metabolic activation in their cytoplasm are either not viable or sometimes enter a dormant state^4^.

Nonetheless, the physical nature of the cytoplasm is poorly understood. The mechanics of reconstituted cytoskeletons purified from the cytoplasm have been studied intensively; strikingly nonlinear responses^5–7^ and slow glassy behaviors^8–11^ have been observed. Apart from cytoskeletons, living cytoplasm is highly condensed, containing a much greater amount [~30% weight fraction^12^] of macromolecules (e.g., globular proteins, ribosomes) that are not tethered to cytoskeletal structures. Although less noticed than the cytoskeleton, this macromolecular crowding also drastically affects cell mechanics^8,13^ and therefore regulates metabolism^14,15^. Physical activation by molecular motors directly induces out-of-equilibrium fluctuations and further alter the mechanics of a living cell’s interior^16^. Glass transition in such activity-driven systems^17–19^ was implied with the anomalous fluctuations observed in living cells^20^. In contrast to thermal fluctuations, however, little is known how to relate such activity-driven fluctuations to the glass formation of the system. The mechanics of living cytoplasm might thus be affected by cytoskeletal networks, metabolic activity, and molecular crowding in a complex manner^20,21^. It is therefore necessary to design an experimental study that dissects out each factor affecting cytoplasmic mechanics and selectively evaluates its consequences.

In this study, we investigated the mechanics in *living* cells by comparing them with several simple *in vitro* models of the cytoplasm that lack cytoskeletons and metabolic activity: a globular biomacromolecule (bovine serum albumin: BSA) solution and three types of cell extracts (*Escherichia coli*, *Xenopus* eggs, and HeLa cells). The mechanics of these models and living cells were studied at the subcellular scale, using the optical-trap-based microrheology (MR) technique^22–29^. Without metabolic activity and cytoskeletons, we found direct evidence that glass transition occurs in these *in vitro* cytoplasm. The viscosity of these materials rapidly increased with the macromolecule concentration. This behavior in a manner of super-Arrhenius^30,31^ is similar to that of fragile colloidal suspensions close to their glass transition. All cell extracts from evolutionary and developmentally distinct cells (bacterial, frog egg, and human cancer cells) showed diverging viscosity at similar critical concentrations *c* ^*^ (~0.34 g/mL) that were surprisingly close to the physiological concentration in *living* cells (~0.3 g/mL).

These observations indicate that the fluidity of metabolism-deficient cytoplasm is “frozen” at physiological concentrations. Regardless of being similar to *c* ^*^, metabolically active living cells (both eukaryotes and prokaryotes) show decent fluidity; however, it is lost in spontaneously inactivated cells. The fluidity of living cells was lost also when macromolecule concentrations were increased by osmotic compression^13^. The manner of the viscosity increase upon crowding was completely different from that in metabolism-deficient cytoplasm. Viscosity of a living cell’s interior retains the Arrhenius-like exponential increase over a wide range of concentrations, that is typical for strong glass formers^8^. These results indicate that living cells are not simply fluidized by their metabolic activity; they still maintain glass-forming property but are driven toward the qualitatively altered state that can be attained in activity-driven systems. We discuss the possible mechanism for the loss of fragility, in relation to the homogenization/relaxation with active stirring in living cells.

## Results

### Mechanics of BSA solutions

Before exploring the cytoplasmic mechanics, we studied a simple model for the crowding of biomacromolecules: a single-component solution of the globular protein BSA which was dissolved in aqueous buffer (HEPES NaCl) homogeneously up to 600 mg/mL^32^. Across this range of concentrations, aggregates or inhomogeneities^33,34^ were rarely observed under high-magnification and high-contrast microscopy.

We performed high-bandwidth passive microrheology (PMR). Thermal fluctuations *u*(*t*) of probe particles (diameter 2*a* = 1 *μ*m) embedded in samples were measured using laser interferometry^35,36^. After application of the fluctuation-dissipation theorem (FDT)^37^ to the power spectral density *P*(*ω*) = ${\int }_{-\infty }^{\infty }$〈*u*(*t*)*u*(0)〉*e* ^*iωt*^ *dt* of the observed fluctuations, the shear viscoelastic properties were obtained: *G*(*ω*) [ = *G*′ (*ω*) + *iG*″ (*ω*); where *ω* is the angular frequency, *G*′ and *G*″ are the storage and the loss moduli, respectively] of the surrounding material. For details, see the protocol in Supplementary Note S1 and our prior study^25^. As detailed later, we found that the BSA solution surrounding the probe did not elastically support the applied stress even at high concentrations. The viscosity *η* of the samples is then given as *η* = $\displaystyle \mathop{lim}\limits_{\omega \to 0}$ *G*″(*ω*)/*ω*. This estimate of viscosity should be equal to the static viscosity at a low shear rate limit^38^.

**Figure 1:**
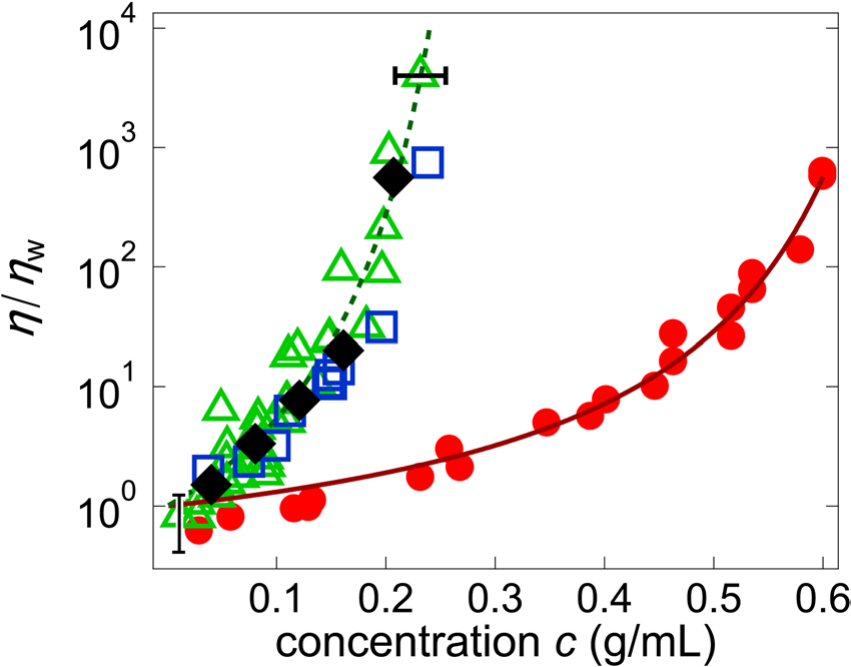
Concentration dependence of viscosity. *η*/*η*_w_ (*η*_w_: viscosity of water) for BSA solutions (red circles) and cell extracts (green triangles: *E. coli*, blue squares: *Xenopus* eggs, black diamonds: HeLa cells). The solid and broken lines are fits of Equation (1) to the BSA and cell extract data, respectively. Error bar in *x*-direction stem from estimation error of the concentration and Error bar in *y*-direction stem from the estimation error of the effect of laser trap. This effect of laser trap on viscosity become negligible when *η*/*η*_w_ become large.

As shown in Fig. 1 (red circles), the static viscosity *η* of the BSA solutions were measured over a wide range of concentrations (0.003–0.60 g/mL). A rapid increase in viscosity was observed above ~0.45 g/mL; this effect does not follow the simple power law or Arrhenius-type exponential behavior. Rather, the Doolittle equation^30,39^ fit the data well:

1 $$\eta /{\eta }_{{\rm{w}}}=\exp \{Ac/({c}^{\ast }-c)\}$$

where *A* = 1.9 and the critical concentration *c* ^*^ = 0.78 g/mL (solid line). Here, *η*_w_ denotes the viscosity of water. Extrapolation of the fit to higher concentrations predicted that *η* would diverge at *c* ^*^ = 0.78 g/mL. The corresponding volume fraction, *φ* ^*^ was estimated to be ~57 % using a partial specific volume of BSA ~7.33 × 10^−3^ mL/g^40^. In the case of hard-sphere colloids, Equation (1) can be interpreted by concept of a free volume that the solid objects can utilize, and which disappears at *c* ^*^^31^. The agreement of our data with Equation (1) suggests that the kinetics of the BSA solutions were arrested upon crowding, primarily because of repulsive interactions between constituent protein molecules^15,41^.

### Mechanics of cell extracts

Next, the mechanics of cell extracts were studied with PMR. We found that intact cell extracts gradually became turbid, and viscosity *η* increased with time when the temperature was increased from 4 °C to ~25 °C (Supplementary Fig. S1, circles). This aging may have resulted from abnormal metabolism in the intact cell extracts, which contain metabolites^42^ but lack factors that ensure normal homeostasis, e.g., membrane proteins^43^. We therefore prepared *in vitro* cell extracts (IVCE) that lack metabolism by removing small metabolites using a centrifugation filter. With this protocol, most proteins and organelles involved in cell mechanics remained in the sample. In the IVCE, the aging-associated increase in viscosity was greatly reduced (Supplementary Fig. S1, triangles). Regardless of the buffers used for IVCEs, their initial viscosity values were similar to corresponding intact cell extracts when measured soon after sample preparation (Supplementary Figs S1 and S2).

We therefore examined the mechanics of IVCEs instead of the intact cell extracts. PMR can be used to obtain viscoelasticity of samples when they are in thermodynamic equilibrium^22,23^. PMR is irrelevant if IVCEs are driven out of equilibrium by residual metabolic activity. If this occurs, probe fluctuations may be much larger than predicted by the FDT. To further complicate this situation, such non-thermal fluctuations eventually become Markov-type random processes if they are monitored for sufficiently long periods^44–46^. Therefore, if PMR alone were carried out, fluctuations out of equilibrium could not be distinguished from those of thermal origin^20,45^. To determine whether the observed fluid-like behavior is induced purely by thermal activation, we carried out active MR (AMR)^25–29^. A sinusoidal force *F*(*t*) = *F*(*ω*) exp(*iωt*) was applied to the probe particles, and the displacement responses *u*(*t*) = *u*(*ω*) exp(*iωt*) were measured. Using the compliant response function, *α*(*ω*) ≡ *u*(*ω*)/*F*(*ω*), we obtained the linear shear modulus of the surrounding medium: *G*(*ω*) [ = *G*′ (*ω*) + *iG*″ (*ω*) = 1/[6*πaα*(*ω*)], where *a* is the radius of probe particles] as detailed in Supplementary Note S1 and in our prior study^25^. Because this protocol does not assume FDT, complete agreement between AMR and PMR confirmed that our samples were in thermodynamic equilibrium (Supplementary Fig. S3).

After these control studies, we measured *η* of IVCEs using PMR. The IVCEs obtained from three different species, *E. coli* (green triangles), *Xenopus* eggs^21^ (blue squares), and HeLa cells (black diamonds), yielded almost the same results, as shown in Fig. 1. The concentration dependence of the viscosity *η* of the *E. coli* extracts fits well Equation (1) (Fig. 1, broken line) at *A* = 3.6 and *c* ^*^ = 0.34 g/mL. Notably, this fit indicates that the critical concentration *c* ^*^ at which *η* diverges was similar to the physiological concentration of macromolecules (~0.3 g/mL or higher)^12^ in *living* cells.

### Fluid-like cell extracts below physiological concentration

We measured frequency-dependent complex shear moduli^25^. The resulting high-bandwidth spectrum (0.1 Hz < *ω* < 100 kHz) also supported the concept of the glassy cytoplasm. For predominantly elastic materials, *G*′ (*ω*) is constant in frequency and greater than *G*″ (*ω*), whereas purely viscous materials have *G*″ (*ω*) which is proportional to frequency [*G*″ (*ω*) ≈ *ωη*] and *G*′ (*ω*) which is negligible. For all BSA solutions as well as IVCEs below 0.2 g/mL, *G*″ (*ω*) was much greater than *G*′ (*ω*) over the entire frequency range measured. This result indicates that the samples behave as a simple fluid, whereas their kinetics gradually slow down upon crowding (Supplementary Fig. S4a–c). In contrast, the IVCE prepared at higher concentrations showed viscoelastic relaxation events at intermediate frequencies; both elasticity and viscosity depended on frequency (Supplementary Fig. S4d).

Similar phenomena have been observed for many colloidal suspensions close to the glass transition^31,38^. When crowded, motion of the unbound macromolecules becomes restricted because an effective cage is formed by the neighboring particles. Below the concentration of glass transition, colloids can still occasionally exit the cage via thermal activation (activated hopping), allowing the overall system to gradually flow. Because of the shortage of the bandwidth in our experiments toward smaller frequencies, the viscosity determined by PMR may be underestimated at concentrations close to *c* ^*^.

**Figure 2:**
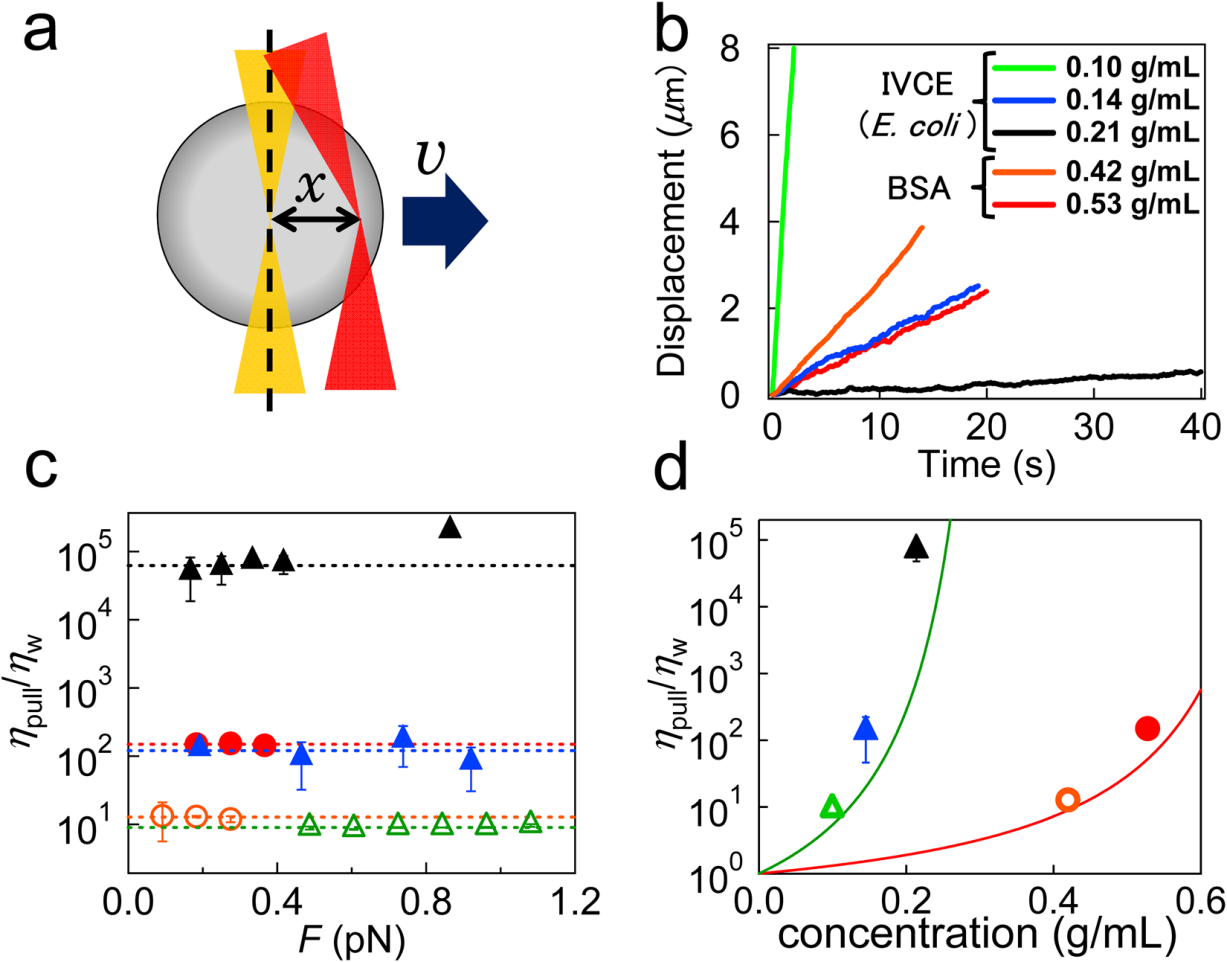
Bead-pulling MR in force clamp mode. (**a**) A schematic of the particle-pulling experiment. A constant force was applied to the probe by maintaining a constant distance *x* between the probe center and laser focus. Resulting velocity *v* yields viscosity *η*. (**b**) The relation between the time and displacement in cell extracts prepared from *E. coli* at various concentrations [0.10 g/mL pulled by 0.25 pN (green), 0.14 g/mL by 0.19 pN (blue), and 0.21 g/mL by 0.14 pN (black)] and in BSA solutions [BSA 0.42 g/mL pulled by 0.18 pN (orange), and BSA 0.53 g/mL pulled by 0.18 pN (red)]. (**c**) The dependence of the applied pulling force on relative viscosity *η*/*η*_w_ in BSA solutions (orange circles: 0.42 g/mL, and red filled circles: 0.53 g/mL) and in *E. coli* cell extracts (green triangles: 0.10 g/mL, blue filled triangles: 0.14 g/mL, and black filled triangles: 0.21 g/mL). (**d**) *η*_pull_ of BSA solutions and IVCEs (*E. coli*) evaluated by particle pulling are plotted together with *η* estimated from PMR (solid curves).

Accordingly, we measured the viscosity of the sample in an alternative way. Optical trapping forces ranging from 0.09 to 1.1 pN were applied to the probe particles, by the feedback control of the laser focus that maintains the constant distance *x* from the center of the probe [force clamp mode, Fig. 2a ^47^]. Using the effective spring constant *k* of the optical trapping potential (therefore referred to as *trap stiffness*), the applied force is obtained as *F* = *kx*. After measurement of the resulting constant velocity *v* of the probe, Stokes’ law provides the viscosity of the surrounding material as

2 $${\eta }_{{\rm{pull}}}=F/6\pi av$$

The viscosity of many biomaterials depends on the applied force *F*. If a material is solidified because of gelation, for instance, it may flow only after “yielding” induced by the failure of stress-bearing structures. For example, probes in crosslinked actin gel stay at rest below yield forces and show occasional jumps and hops because of intermittent yielding when they move (Supplementary Fig. S5). Probes in our sample (BSA solutions and cell extracts), however, flow even if *F* is much less than 1 pN. In addition, we did not observe any indication of intermittent yielding in the probe trajectory (Fig. 2b). All data, except for those from the concentrated IVCE (0.21 g/mL), showed constant viscosity (*η*_pull_) in *F* (Fig. 2c). Such linear behavior is called Newtonian and is expected for simple liquids^48^. Although the concentrated IVCE (0.21 g/mL) showed shear thickening (increased viscosity during shear) at greater *F* (>0.4 pN), Newtonian behavior was also observed at a smaller *F* (<0.4 pN; *F*ig. 2c). Thus, all IVCEs behaved as a Newtonian fluid when the applied stresses were sufficiently small. The evaluated *η*_pull_ is plotted in Fig. 2d and was consistent with *η* determined by PMR (indicated with solid lines) although *η*_pull_ was slightly greater than *η*, particularly when the sample concentration was close to *c* ^*^.

### *In vitro* cytoplasm similar to glassy suspensions

The jamming volume fraction φ* ≈ 57% was also reported for eye lens *α*-crystallin^41^. It is reasonable that tightly folded globular proteins (BSA and *α*-crystallin) show jamming at the volume fraction similar to hard spheres (φ* ~ 64%)^31,49^. A slight difference in φ* as compared to hard spheres was expected because φ* can weakly depend on the shape and polydispersity of colloids^50,51^. In contrast, it remains unclear why IVCEs showed much smaller *c* ^*^ values than did tightly folded proteins or hard spheres. In the cytoplasm, macromolecules assemble into higher-order structures which contain hydrated water. The situation is similar to suspension of dilated hydrogel particles that vitrify at extremely small concentrations of macromolecules^52^. The effective volume fraction *φ* in IVCE must be higher than the estimate from the dried mass fraction. This phenomena may explain the apparent discrepancy in *c* ^*^ between BSA and IVCE.

**Figure 3:**
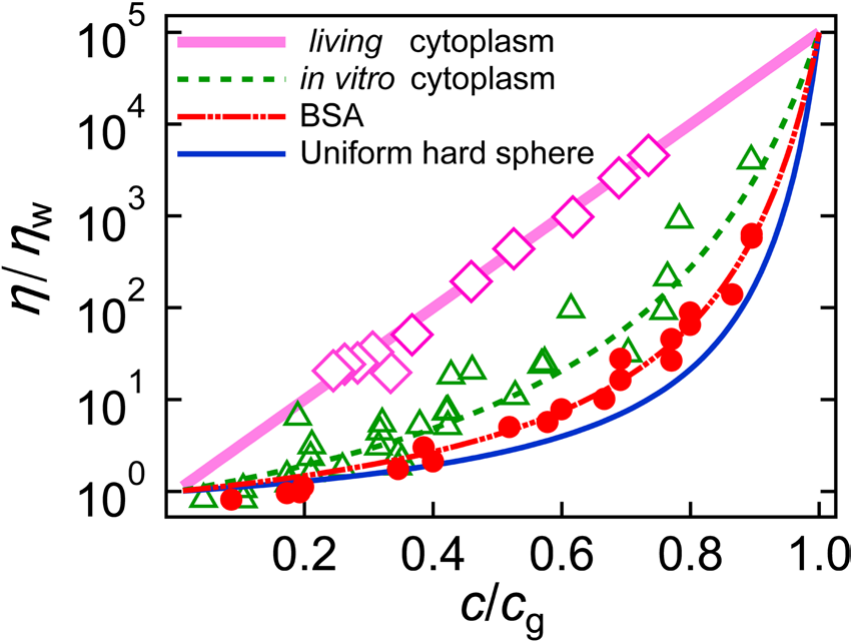
Angell plots. Relative viscosity *η*/*η*_w_ as a function of scaled concentration *c*/*c*_g_ for BSA (red circles and the dash-dot-dot curve), for cell extracts from *E. coli* (green triangles and the dotted curve), and for cytoplasm in a living cell (pink diamonds and the solid line). Curves are the fits of Equation (1); *c*_g_ for each sample was determined as a concentration where *η*/*η*_w_ becomes 10 ^5^-fold greater than that in water. The solid curve represents the results reported in refs^31,39^ for the suspension of hard-sphere colloids of uniform size. The solid line indicates Arrhenius behavior for strong glass to which viscosity in living cells conforms.

In order to further explore the analogy between cell extracts and soft glassy colloids, we formatted our viscosity data (BSA and cell extracts) as an Angell plot^53^ (Fig. 3). The abscissa of the plot was scaled to the glass transition concentration *c*_g_, which is defined as the concentration where *η* becomes 10^5^ times greater than that in water^50^. Fragility, an important feature of glass transitions, became evident in this plot. “Fragile” glass formers change their viscosity rapidly when they are close to *c*_g_. Strong glass formers, in contrast, constantly change their viscosity over the whole range of the plot (the Arrhenius-like straight line in Fig. 3). Fragility is therefore defined as the logarithmic slope of the scaled graph at the glass transition *c* → *c*_g_^54^. For colloidal suspensions, it has been shown that “fragility” reflects local interactions and physical properties of each colloid, such as stiffness and size dispersion^50,51^. Stiffer and uniform colloids show high fragility, for instance. Angell plots of our data and data from hard-sphere suspensions [reported in refs^31,39^, solid line] revealed the magnitude of fragility that is hard-sphere colloids > BSA > IVCEs. Given that the components in cell extracts are softer and more polydispersed than BSA or hard spheres are, our experimental results support the idea of glass-forming cytoplasm.

### GFP diffusion in *in vitro* cytoplasm and in *living* cells

**Figure 4:**
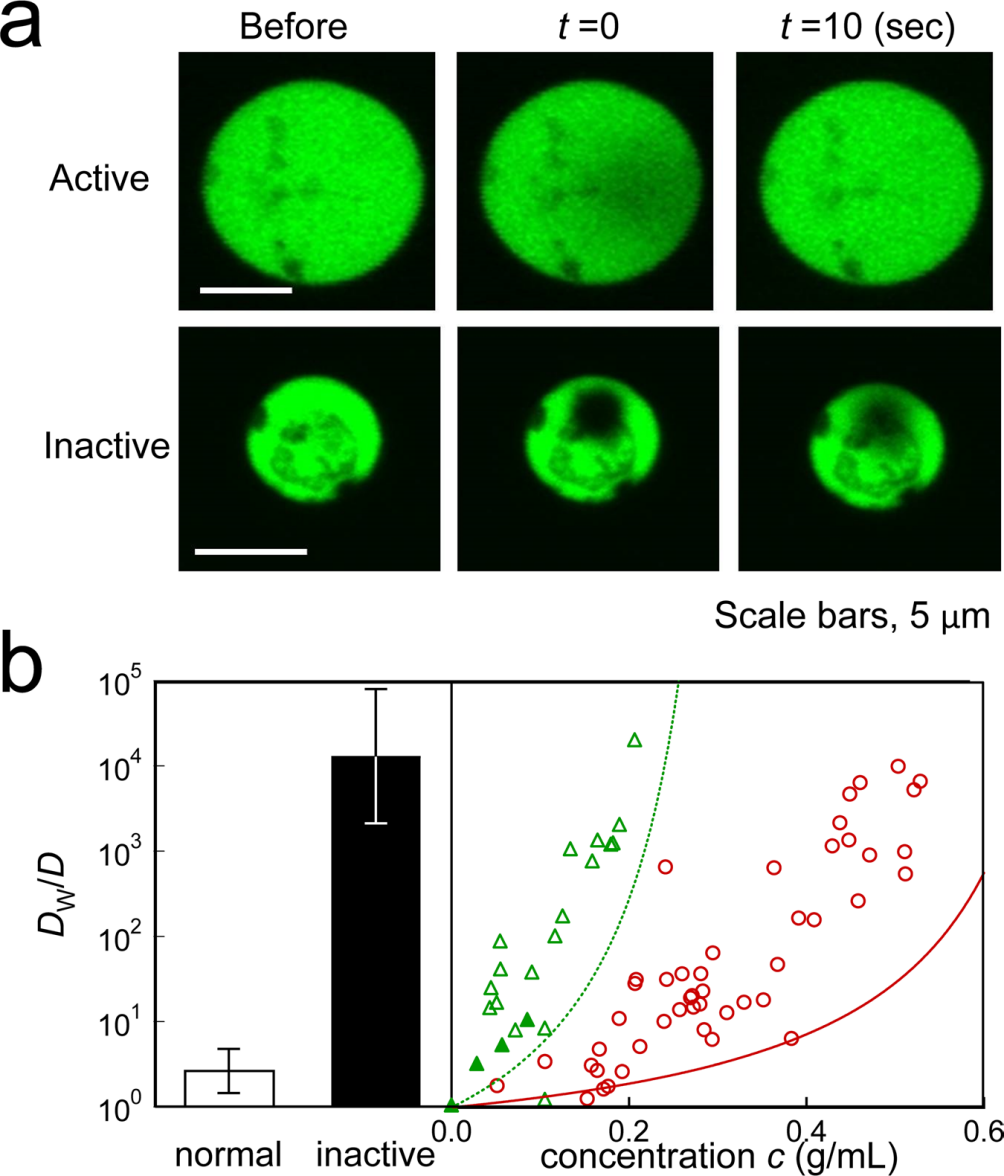
Diffusion in *living* cytoplasm and *in vitro* cytoplasm measured by FRAP. (**a**) Fluorescent images of GFP-labeled living spheroplasts of *E. coli* before, immediately after (*t* = 0 s), and 10 s after photobleaching. The photobleached region in a metabolically inactive spheroplast remained dark even at 10 s after photobleaching (lower panels), whereas the dark region was obscured already at *t* = 0 in normal spheroplasts (upper panels) because of rapid transport. (**b**) Inverse of diffusion coefficients *D* of GFP in IVCEs/liposomes (open triangles), IVCEs/emulsions (filled triangles) and BSA/liposomes (circles) measured with FRAP. These data were normalized to the value in water as *D*_w_/*D*. The broken and solid curves indicate *η*/*η*_w_ in IVCEs and BSA solutions which are measured by PMR (same lines with that in Fig. 1), respectively. *D*_w_/*D* of GFP in normal (white bar) and metabolically inactive spheroplast cells (black bar) are also shown.

Our study indicates that IVCEs at the physiological concentration (~0.3 g/mL) were on the verge of glass transition; simple extrapolation of the fit in Fig. 1 gives *η* of IVCE that is higher than 10^8^ [Pa ⋅ s]. Here, the concern was whether the cells were viable with the virtually solidified cytoplasm where efficient transport of molecules was stopped. We therefore investigated “apparent” diffusion of fluorescently labelled macromolecules (GFP) in living cells. We first chose giant protoplasts of *E. coli*, which were artificially grown to spherical shape by digesting the cell walls with lysozymes (diameter ~10 *μ*m; Supplementary Fig. S6a). Transport of the GFP molecules (expressed in spheroplasts) towards the photo-bleached regions was examined by epifluorescence microscopy (FRAP, see experimental section). In most spheroplasts, the fluorescence of GFP almost immediately recovered soon after photo-bleaching (Fig. 4a). Some *E. coli* cells (~1%) ceased growing after the cell wall was digested. Such minor spheroplasts showed irregularly heterogeneous textures under an optical microscope (Supplementary Fig. S6b). In FRAP experiments, the photo-bleached region in these minor spheroplasts was retained even 10 s after the brief exposure of photo-bleaching light (Fig. 4a). Reportedly, in a recent study, molecular transport in the bacterial cytoplasm strongly depends on metabolic activity^20^. These minor spheroplasts might be then metabolically inactive^55^.

As a control, we encapsulated IVCEs into spherical bags (liposomes and emulsions) together with GFP and measured its diffusion by FRAP. We compared the inverse of the diffusion of GFP (Fig. 4b, triangles and circles) and the viscosity *η* measured by microrheology (solid line and broken line are the same as those in Fig. 1), after normalizing both with the values measured in aqueous buffer (*D*_w_ and *η*_w_). For the small protein GFP, cytoplasm in the surrounding medium is not regarded as a continuum. Hence, the Stokes-Einstein relation *η* ∝ 1/*D* may not hold. Furthermore, the unavoidable errors enter the estimate of concentrations when cytoplasm is encapsulated in liposomes. We could still say that diffusion of both BSA solution and IVCE showed trends consistent with those obtained by microrheology; diffusion of GFP decreases rapidly toward the glass transition point.

In contrast, the “apparent” diffusion of GFP in normal living spheroplasts [*D*_w_/*D* ≈ 2.7 (white bar in Fig. 4b)] was at least several orders of magnitude greater than diffusion in IVCEs (Fig. 4b, triangles and broken curve) and in abnormal inactive spheroplasts (*D*_w_/*D* ≈ 13000; Fig. 4b, black bar). In living cells, the long-term fluctuation of embedded probes is enhanced because of the out-of-equilibrium metabolic activity^44^. In addition to thermal forces, active forces generated by molecular motors also drive the diffusion of macromolecules in cells^45,56^. That is the reason the term “apparent” is used for the diffusion in living cells. Research into the out-of-equilibrium fluctuations in living materials is the current focus in the field of biological physics. However, merely observing slow fluctuations (“apparent” diffusion) of molecules is not sufficient to study their relation to glassy dynamics because fluctuations in such activity-driven glasses have been rarely explored and remain elusive in the field of glass studies to date.

### High-bandwidth microrheology in eukaryotic cells

Even if slow fluctuation in cells is dominated by the out-of-equilibrium activity, we confirmed that the FDT is satisfied at high frequencies by comparing AMR and PMR (Supplementary Fig. S7) as reported in prior studies^45,57,58^. We therefore carried out high-bandwidth microrheology to observe high-frequency fluctuation in living cells (HeLa, MDCK and NIH3T3) using colloidal probe particles [melamine particles coated with polyethylene glycol^59^, diameter 2*a* = 1 *μ*m] incorporated into the cell interior (Supplementary Note S1, Fig. S8 and Methods). The power spectral density of probe fluctuations *P*(*ω*) at high frequencies is converted to the imaginary part *α*″ of the response function *α*(*ω*) based on the FDT [*P*(*ω*) = 2*k*_B_ *T α*″/*ω*] (Supplementary Fig. S7a and Supplementary Note S1). It was crucial to implement feedback technology in order to smoothly track the actively fluctuating probes in cells^60^.

We found that *in vitro* cytoplasm in the crowded condition (>0.2 g/mL) and *living* cells in confluent epithelium (HeLa, MDCK) share a similarity in their mechanics; both showed the same power-law form of frequency dependence at high frequencies, i.e., *G* ~ (*i ω*)^0.5^ or equivalently *α* ~ 1/*G* ~ (*i ω*)^−0.5^ (Supplementary Figs S4d and S9). The same power-law dependence has been observed for densely packed emulsions and swollen-gel colloids in their glassy state^61^ and theoretically articulated as the glassy relaxations typical for densely packed soft colloids with a slippery interface^24,61^. In prior studies, cellular mechanics have been explored by poking cells from the outside^8,62–64^, which were likely affected by the cell cortex cytoskeleton *G* ~ (*i ω*)^0.75^ rather than the cytoplasm *G* ~ (*i ω*)^0.5^.

In order to change concentrations of intracellular macromolecules (0.2–0.6 g/mL), the osmotic pressure was changed by adding sucrose or water to the culture media^8^. The volume fraction of intracellular macromolecules was estimated from the ionic strength of the media according to Ponder’s law^8,65,66^. Notably, fluctuations of the probe particle responded immediately to the change of osmotic pressure (Supplementary Fig. S10). We estimated the viscosity *η*_@4kHz_ ∝ 1/*α*″_@4kHz_. Here, @4 kHz in the subscript indicates the value at 4 kHz.

**Figure 5:**
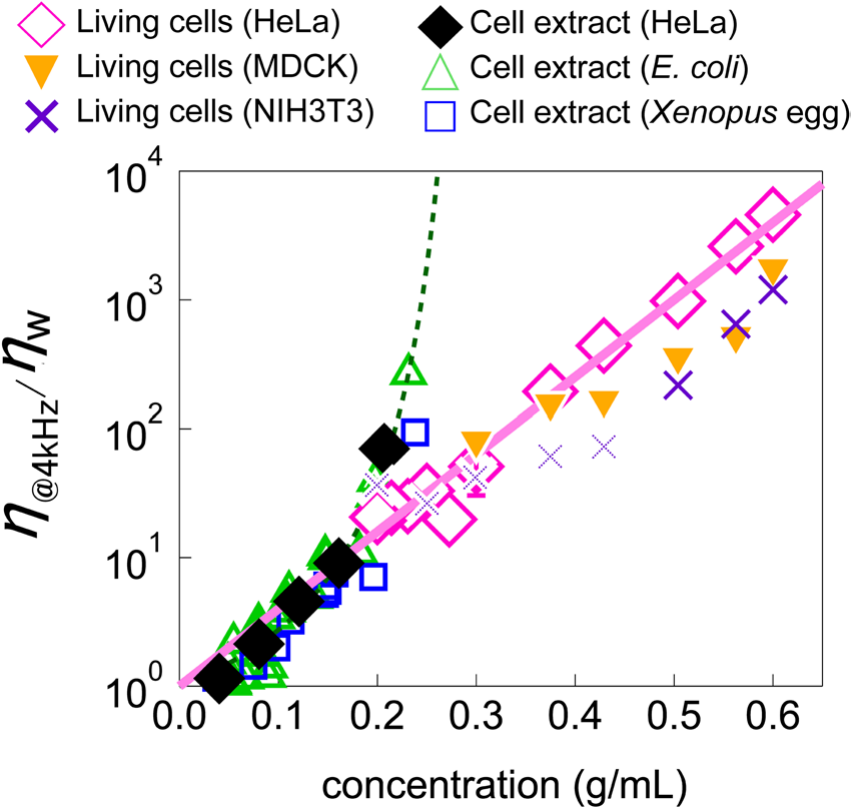
Concentration dependence of viscosities at 4 kHz in living cells and in cell extracts. *η*_@4kHz_/*η*_w_ for cell extracts (green triangles: *E. coli*, blue squares: *Xenopus* eggs, black diamonds: HeLa cells) and for cells (pink diamonds: HeLa cells, filled orange downward triangles: MDCK cells, purple crosses: NIH3T3 cells). The green solid line represents fit of Equation (1) to *η*_ext@4kHz_/*η*_w_ measured by PMR. A solid pink line is the exponential fit for cell extracts at low concentration and living cells.

Concentration dependence of relative viscosity in *living* cells *η*_cell@4kHz_/*η*_w_ is plotted in Fig. 5 (open pink diamonds: HeLa, filled orange downward triangles: MDCK, purple crosses: NIH3T3) and is compared to that of *in vitro* cytoplasm (*η*_ext@4kHz_/*η*_w_). We found another typical glass-forming behavior that *η*_cell@4kHz_/*η*_w_ increased exponentially with the macromolecule concentration. This result indicates that the *living* cell interior can be categorized as a strong glass former^50^. Angell plots of *η*_cell@4kHz_ revealed Arrhenius-like behavior, which is evidently different from *in vitro* cytoplasm (Fig. 3). HeLa (highly malignant cancer) and MDCK (epithelial) form tightly-packed monolayers which have less cytoskeletons except membrane peripheries whereas NIH3T3 cells prefer to be isolated and takes flattened morphology which is rich in actin cytoskeletons^67^. Also, it is known that cancer cells systematically soften during the progress of malignancy^68^. Intracellular viscosity of all these different cells, however, shows quantitatively similar exponential increase though *η*_cell@4kHz_/*η*_w_ in NIH3T3 cells seems to slightly deviate at low concentrations (0.2–0.43 g/mL). Since the power-law exponents *β* of the form *α* ∝ *ω* ^−*β*^ in NIH3T3 cells are distinct from 0.5 at low concentrations (Supplementary Fig. S11), NIH3T3 data at low concentrations (0.2–0.43 g/mL) might be affected by cytoskeletons.

The same tendency in static “elasticity” of cell cortex was reported in ref.^8^, and its underlying physics was discussed in relation to the deformability of constituent biomacromolecules; soft colloids generally form strong glasses upon crowding^50^. Notably, the “viscosity”, which we measured in cells, more directly manifests the critical slowing down of relaxations in glass-forming process. Non-fragile behavior in viscosity of *living*-cell interior extends toward concentrations much higher than physiological values, where *in vitro* cytoplasm already comes to jamming (Fig. 5). Extrapolation of Arrhenius-like behavior of *η*_ext@4kHz_ at small concentrations (below 0.2 g/mL) toward higher concentrations overlaps with that of living cells, *η*_cell@4kHz_/*η*_w_, as shown with the eye guide given by the thick pink line.

## Discussion

We found that the glass-forming behavior of IVCEs and in *living* cells are quantitatively universal, which were not expected prior to this study (Figs 1 and 5). Chemical details of intracellular components of somatic vertebrate cells are largely different from those in prokaryotic cells (*E. coli*) as well as in germ cells (*Xenopus* egg)^1,69^. Constituents of macromolecules in *living* cells dynamically change during the cell cycle^70^ and at different stages of embryonic development^71^. If cytoplasm was solidified because of gelation with specific cross-linking (hydrogen or covalent bonding that require specific pair of molecules), its mechanics would largely depend on the slight difference of these chemical constituents. On the other hand, repulsive interactions that govern colloidal glass transitions are generally non-specific, and are affected by physical properties of colloidal constituents such as their shape, size, and stiffness, rather than their chemical details^38^ (Supplementary Figs S12 and S13). Possibly, dispersion of these physical nature of cytoplasmic constituents does not vary much for different cell types, that can explain the observed universality in the glass-forming behaviors of *in vitro* and *living* cytoplasm.

In recent years, the concept of the jamming phase diagram has been proposed, highlighting the significance of mechanical forces for the behavior of glass-forming materials^72^. For dense colloidal suspensions, thermal hopping away from the cages formed by surrounding colloids is suppressed upon crowding. The system is fluidized if the energy injected by the mechanical loading instead activates the hopping process. The steady shearing of the crowded suspension, for instance, greatly reduces its viscosity to 1/1000 or less^73^ (referred to as *shear thinning*). The FDT violation and super diffusion seen in Supplementary Fig. S7 clearly show that non-thermal forces are present in living cells. In cells, forces are likely generated more randomly on a smaller length scale by e.g. motor proteins. In this sense, the role of metabolic activities in reducing cellular viscosity can be qualitatively similar to that of temperature and/or mechanical loading in dense colloidal suspensions; the structural relaxation is enhanced by non-thermal activity^17–19^. Note that not only the IVCEs but inactive cells are glassy (or less fluidized at least) at biologically-relevant concentrations (Fig. 4b). We found that the glass transition points of IVCEs correspond to physiological concentration of living cells. For efficient biochemical reactions, both concentrated situations and efficient transportation of materials are preferred. These requirements would conflict in non-living systems, especially for fragile glasses. It is then reasonable for living cells to regulate their concentrations close to the glass transition point for IVCEs and thereby simultaneously satisfy these requirements.

Non-thermal activation is the key to understand the difference of fragility of cell extracts and in living cells (Fig. 3). Being subjected to non-thermal activation in excess of thermal one, free volume that macromolecules in living cells can explore should be larger than that in *in vitro* cytoplasm; the point of jamming *c* ^*^ can shift to higher concentrations^72^. It is also known that the fragility of colloidal glass formers correlates with the slope of the *E* ≡ [elastic energy per colloids (stiffness of colloids)]/[activation energy] as a function of colloidal concentration; a greater slope leads to fragile glass formers^50^. Activation is solely thermal in fragile IVCEs whereas non-thermal activations in living cells make the slope (*dE*/*dc*) smaller. Without non-thermal activation, colloidal glasses are strong only when constituent colloids can be compressed more than 10-fold in their volume during glass formation^50,74^. Such extreme deformability is unlikely for a large part of cell constituents such as ribosomes. Therefore, the softness of cell constituents alone does not explain the non-fragile behavior of living cells; the metabolic activity is essential.

It is recently acknowledged that fragility might be related to the dynamic heterogeneity; more fragile glasses tend to have larger dynamic heterogeneity^75,76^. When *dE*/*dc* is large, colloids in suspension cannot relax the frustration in local arrangements independently. This leads to inhomogeneous distributions of residual forces and stresses in the sample which can relax only through rare cooperative rearrangements^77^. On the other hand, soft gels, emulsions and foams form strong glasses^50^. Even if they are packed, they can still locally relax that the heterogeneous dynamics might be less likely to be observed. In our experiments, heterogeneity of viscosity in cell extracts were not large (Supplementary Fig. S14). Likely, the characteristic length of the heterogeneity is smaller than probe sizes (Supplementary Fig. S15). Also, we speculate that heterogeneity in living cells were remarkably smaller than expected^20^ since cell interior is actively stirred and homogenized by non-thermal activation (Supplementary Fig. S7). Here, active stirring in cells can relax the residual stresses that would convert the fragile IVCEs to strong glass formers.

Direct evidence was obtained for the glass-forming behavior of *in vitro* cytoplasm and *living* cytoplasm, by measuring their mechanical properties using particle-tracking and manipulation technique. It was crucial to explore the mechanics of simplified *in vitro* model cytoplasm from which effects of cytoskeletal networks and cell metabolism were artificially removed, and to compare them with those in living cells. We found that the mechanical properties of the model cytoplasm were identical to those known for fragile colloidal glass formers that steeply increase their viscosity close to glass transition. They showed well-defined Newtonian viscosity below the physiological concentration of living cells (~0.3 g/mL). The critical concentration *c* ^*^ for jamming was found to be nearly identical to the physiological concentration of metabolically active cells. In contrast, microrheology performed in living cells revealed finite fluidity though it is reduced in spontaneously deactivated cells. Viscous modulus in living cells was also increased by the increased macromolecular concentration via osmotic compression, however, more gently in a manner of complete Arrhenius typical of strong glass formers. All these observations support that the *non-living* cytoplasm at the physiological concentration is on the verge of glass transition. The *living* cytoplasm gains some fluidity via metabolic activity by changing its glass-forming characters from that of fragile to that of strong glass formers.

## Methods

### Experimental setup for MR

**Figure 6:**
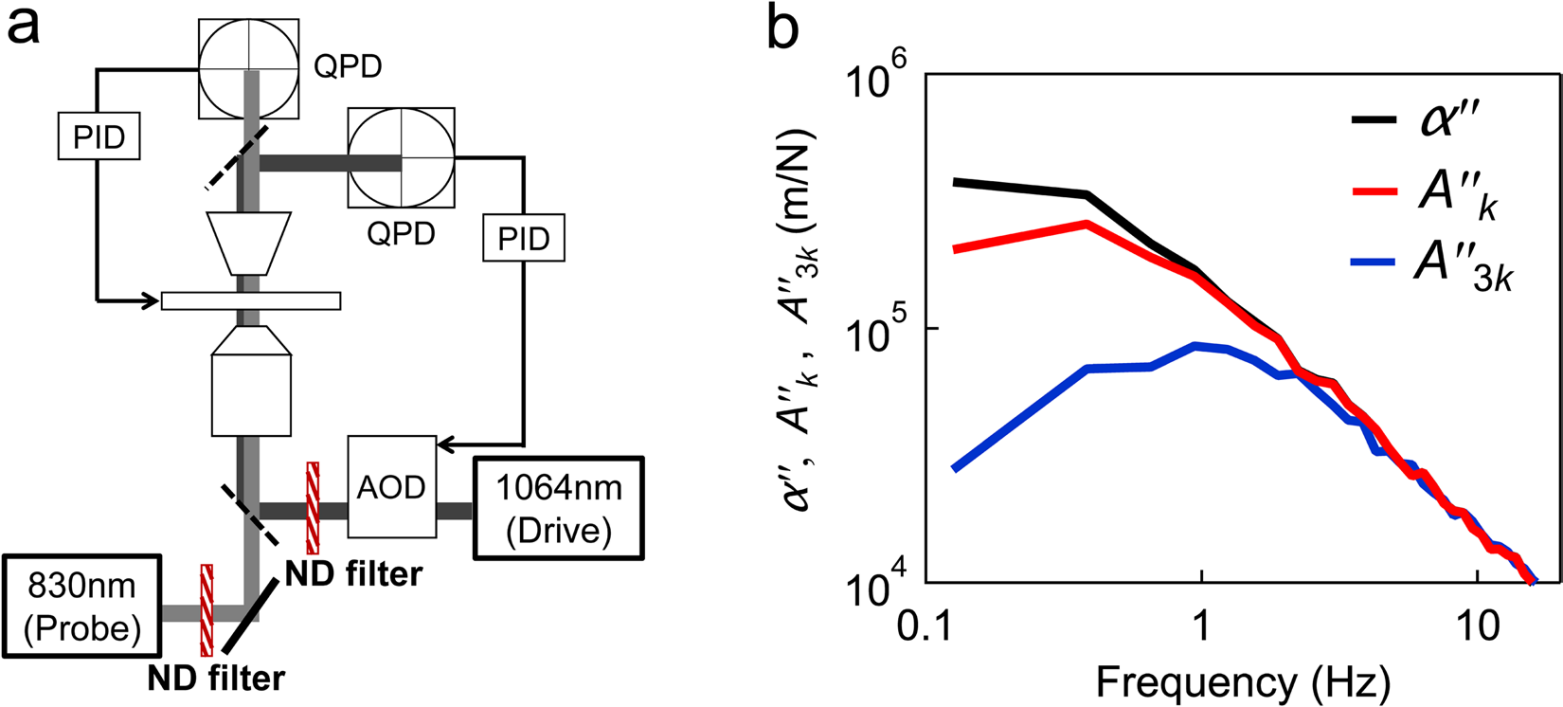
The microrheology setup and trapping-force calibration. (**a**) A simplified schematic of the experimental setup. A constant trapping force was applied by rapidly steering the position of the drive laser (*λ* = 1064 nm) using the feedback-controlled AOD (force clamp). Displacements of the probe were detected using a fixed probe laser (*λ* = 830 nm). In the bead-pulling experiments, the piezo stage was also feedback controlled in order to eliminate the probe drift. (**b**) Imaginary parts of the response functions, *A*_*k*_ (red curve), *A*_3*k*_ (blue curve), and *α* (black curve), measured in BSA solution (0.54 g/mL). Full complex responses including real parts are shown in Supplementary Fig. S10. With the increase of trap stiffness, the responses were suppressed at low frequencies.

Details of the MR using the optical trap and laser interferometry technique are given elsewhere^23–25,36^ (Supplementary Note S1). The experimental setup consists of two optical traps made from lasers with two different wavelengths (drive and probe laser; Fig. 6a). Our setup additionally incorporates 3-dimentional feedback of a piezo-mechanical sample stage (Nano-LP200, MAD CITY LABS INC., WI, USA) that enables a probe particle to be stably tracked in vigorous flows in cells^60^. Briefly, the output *V*(*t*) of the quadrant photodiode (QPD), which is proportional to probe displacements *u*(*t*), was used for the PID feedback of the piezo-actuated stage that maintains the particle in the center of the laser focus. The total displacement of the probe particle in the sample was obtained as the sum of the piezo displacement *u*_stage_ and the displacement of the probe particle from the laser focus *u*_QPD_. PMR in living cells and the particle pulling were both performed under the feedback.

### Particle pulling with a force clamp

In order to stably apply an extremely small force (~0.1 pN) to the probe particle, we used another feedback technique known as a force clamp^47^. The schematic of this pulling method is shown in Fig. 2a. QPD output for the drive laser is fed to an analog PID controller (SIM960; 100 kHz, Stanford Research Systems, Inc., Sunnyvale, CA, USA) that creates a feedback signal to control an acousto-optic deflector (AOD (model DTSX-400-1064, AA Sa, Orsay, France)), and thus the distance of the drive laser focus from the bead center is kept constant. Using this technique, constant force was applied even if the probe particle fluctuates. Under the constant force application, the probe bead gradually moves in one direction that was tracked by the feedback of the piezo stage as explained above. The total displacement of the probe particle including the driven drift motion was obtained as *u* = *u*_QPD_ + *u*_stage_.

### Calibration of laser interferometry experiments

For microrheology experiments using the optical-trap and laser-interferometry techniques, diffraction of the laser due to the displacement of a trapped particle was detected using a quadrant photodiode^36^ (Fig. 6a). The output *V*(*t*) of the QPD must be appropriately calibrated to probe displacements *u*(*t*) and optical-trapping forces *F*(*t*). The conventional method of calibration is to measure the Brownian motion of the same probe particle in a pure solvent^35^. Comparing the observation and theoretical prediction based on FDT, calibrations for both displacements and trapping forces were attained simultaneously. This protocol is valid when the difference in the refractive index between sample solutions and a pure solvent is negligible, i.e., for dilute samples.

### Calibration for probe displacements

The calibration method described above is not valid for the concentrated samples used in this study. We therefore obtained the calibration factors for probe displacements *Cal* ≡ *u*(*t*)/*V*(*t*) directly *in situ* for every particles. A probe particle was trapped using a drive laser and oscillated slowly at 0.5 Hz. *Cal* for the probe laser was obtained by comparing the output *V*(*t*) of the QPD for the fixed probe laser and *u*(*t*) obtained from the video of the probe particle (Supplementary Fig. S8). In the case of the drive laser, the piezo stage was sinusoidally oscillated, and the resulting *V*(*t*) and *u*(*t*) were compared in the same manner.

### Calibration of trap stiffness

A new calibration method (for trap stiffness) that is applicable to concentrated cytoplasmic models was employed in this study. A particle in an optical trap is confined within a harmonic potential created by the trapping laser. Therefore, optical trapping affects both the thermal motion and the response of the particle to the applied force. Trap stiffness *k* is determined as *F*/*x*, where *F* is the trapping force and *x* is the distance between the probe center and the laser focus. We describe here the response function of the probe that is in the trap with stiffness *k* as *A*_*k*_(*ω*) and the response in the absence of the optical trap as *α*(*ω*). The response in trap *A*_*k*_(*ω*) was suppressed as compared to *α*(*ω*), as shown in Figs 6b and S16, at low frequencies. We then measured another response function *A*_n*k*_(*ω*) by increasing the laser strength by n-times (Fig. 6b). As shown in Fig. 6b, further suppression of the response function was observed at low frequencies, clearly because the trap was threefold stronger. The relation among *A*_*k*_(*ω*), *A*_n*k*_(*ω*), and *α*(*ω*) is given as follows^25^:

3 $$\alpha (\omega )=\frac{{A}_{k}(\omega )}{1-k{A}_{k}(\omega )}=\frac{{A}_{{\rm{n}}k}(\omega )}{1-{\rm{n}}k{A}_{{\rm{n}}k}(\omega )}$$

The trap stiffness was obtained as

4 $$k=\frac{{A}_{k}(\omega )-{A}_{{\rm{n}}k}(\omega )}{({\rm{n}}-\mathrm{1)}{A}_{k}(\omega ){A}_{{\rm{n}}k}(\omega )}$$

from the second equality in Equation (3).

### Sample preparations

For BSA solutions, 100 mg of BSA (Sigma-Aldrich, St. Louis, MO) was dissolved in 1 mL of NH buffer (100 mM NaCl and 20 mM HEPES-KOH, pH 7.6). The samples were washed with a 10-fold volume of NH buffer using a 50-kDa filter unit (Amicon Ultra-15, Merck-Millipore, Darmstadt, Germany) at 4 °C. After four washes, the samples were concentrated to ~600 mg/mL by centrifugation with a 3-kDa filter at 4 °C.

The cytoplasm of *Escherichia coli*, HeLa cells, and *Xenopus* eggs were extracted as given in Supplementary Note S2. To inhibit cytoskeleton polymerization, 0.1 mM cytochalasin B was present throughout the sample preparation in the cytoplasm of *Escherichia coli*, HeLa cells, and *Xenopus* eggs. Before experiments, cell extracts (IVCE) were diluted with a 10-fold volume of NH buffer and washed by centrifugal filtration at 4 °C using a filter with a 3-kDa molecular cut-off (Amicon Ultra-15, Merck-Millipore). After washing 4 times, the samples were concentrated by further centrifugal filtration. The concentration of the resulting IVCE was determined using a Bradford assay. We assumed that the efficiency of the assay and the ratio of proteins to total macromolecules were constant for different concentrations of IVCEs taken from each cell species. The results of the assay were calibrated to the actual concentrations of macromolecules by measuring the dry weight of the stocks. In IVCE obtained from *E. coli*, for instance, approximately 1.5-fold more biomacromolecules than proteins (as evaluated by Bradford assays) were present in the final solutions. The ratio of contents obtained in this study was consistent with that in other studies on the ratio of proteins to RNA in *E. coli* ^12^. All experiments using *Xenopus* were approved by the Institutioonal Animal Care and Use Committee of Kyushu University (Fukuoka, Japan) and carried out in accordance with the guidelines set by this committee.

Giant spheroplasts of *E. coli* were prepared as described elsewhere^43^ (Supplementary Note S2). HeLa, MDCK and NIH3T3 cells were cultivated on fibronectin-coated glass-bottom petri dishes in Dulbecco’s modified Eagle’s medium (DMEM) with 1 mg/mL of glucose, 100 units/mL of Penicillin, 0.1 mg/mL of Streptomycin, 2.5 *μ*g/mL of Amphotericin B, and 10% fetal bovine serum at 37 °C. When the population of cells in a flask was confluent, the medium was replaced by a CO2-independent culture medium (Wako). The probe particles (melamine particles coated with polyethylene glycol brush, diameter of 2*a* = 1 *μ*m, and refractive index of 1.68, Microparticles GmbH) were introduced into the cells using a Gene gun (PDS-1000/He, Bio-Rad, Hercules, CA, USA)^78^. The dishes were then incubated at least for several hours until the cell membranes recovered. Excess beads were removed by washing the dishes with phosphate-buffered saline. Finally, a CO2-independent culture medium without serum was added to perform the measurements.

### FRAP analysis

Liposomes containing concentrated BSA and IVCE were prepared using the droplet-transfer method^79,80^ with 1 mM eggPC in mineral oil (Nacalai Tesque, Kyoto, Japan, product code: 23306-84). Initial macromolecule concentrations of BSA and IVCE were 0.14 and 0.11 g/mL in NH buffer (220 mosM), respectively. After formation, liposome solutions were mixed with equal volumes of 220, 660, and 1100 mM aqueous sucrose in order to osmotically compress and concentrate the contents. Macromolecule concentrations inside the liposomes were estimated from the fluorescence intensity of GFP (0.2 mg/mL) measured by confocal microscopy (Olympus FV1200, Tokyo, Japan). After photobleaching with a circular spot of a radius *r* ~ 1 *μ*m for 1 s at full laser power, the fluorescence recovery was recorded. For convenience, the recovery intensity as a function of time was fitted to the following conventionally used equation:

$$f(t)=\exp \,(-2t/t)[{I}_{0}(2t/t)+{I}_{1}(2t/t)],$$

where *I*_0_ and *I*_1_ indicate modified Bessel functions, and *τ* is the characteristic time scale of diffusion^81^. This formula is merely valid for 2D diffusion, typically when photobleaching was performed with an unfocused laser in bulk solution. The formula *r* ^2^/4 *τ* then gives the diffusion coefficient of GFP. In this study, however, FRAP was performed with a focused laser in samples with limited dimensions (spheroplasts and liposomes). Because the measured value of *r* ^2^/4 *τ* merely reflects the scale of the diffusion in this case, it is crucial to normalize it to the value measured for pure solvents with all other conditions unchanged.

## Electronic supplementary material

Supplementary Information
