# Supplementary material for: Universal glass-forming behavior of in vitro and living cytoplasm
Source: Sci Rep. 2017 Nov 9;7:15143. doi: 10.1038/s41598-017-14883-y (PMC5680342; doi:10.1038/s41598-017-14883-y)
Supplement: Supplementary file 1 — Supplementary Information [file 41598_2017_14883_MOESM1_ESM.pdf]

Supplementary Information

Universal glass-forming behavior of *in vitro* and *living* cytoplasm

Kenji Nishizawa, Kei Fujiwara, Masahiro Ikenaga, Nobushige Nakajo, Miho Yanagisawa, Daisuke Mizuno

Note S1. Passive microrheology (PMR) and Active microrheology (AMR)

Experimental setup for MR

For PMR, the thermal motions of the probe particles were detected by laser interferometry using a probe laser (λ = 830 nm, IQ1C140, Power Technology, Alexander, AR, USA). The output signal from a quadrant photodiode (QPD) was digitized at 100 kHz with an A/D board (AD24 DSA board PCI-4462, National Instruments, Austin, TX, USA) and recorded on a personal computer. For AMR, the other laser (λ= 1064 nm, Nd: YVO4, Compass, Coherent, Santa Clara, CA, USA) was operated at higher power as the drive laser. The optical trapping force was applied in a controlled manner by rapidly steering the location of the laser focus using an acousto-optic deflector (AOD (model DTSX-400-1064, AA Sa, Orsay, France). The resulting motions of the probe particle were detected using the probe laser.

Passive microrheology (PMR)

As written in detail in ref. 25 in the main text, thermal fluctuations of probe particles were observed in order to obtain mechanical properties of surrounding media (cell extracts and protein solutions). Micrometer-sized probe particles (08226 Polysciences, Inc., polystyrene latex, radius a = 0.5 μm and MF-R-S1141, micro particles GmbH, melamine resin particles, radius a = 0.57 μm) were dispersed in sample solutions and trapped by a probe laser (λ = 830 nm). The transmitted laser was collected by the condenser lens. Laser was slightly diffracted due to the displacements of the probe. The diffracted pattern at the back-focal-plane of the condenser lens was projected on a quadrant photodiode (QPD). The voltage signals $V(t)$ of the QPD, which are proportional to the probe displacements, were recorded in PC. The displacement of a probe particle $u(t)$ is calculated as $u(t) = Cal\ V(t)$ where Cal is the calibration factor, directly determined for each probe particle as shown in Fig. S8. The power spectral density $P(\omega) = \int_{-\infty}^{\infty}\langle u(t)u(0)\rangle e^{-i\omega t}$ of the probe fluctuation is related to imaginary part of the response function of the probe $A''_k(\omega)$ as $P(\omega) = 2k_B T A''_k(\omega)/\omega$ via the fluctuation-dissipation theorem (FDT) as given in ref. 37 in the main text. $A_k(\omega) = A'_k(\omega) - iA''_k(\omega)$ represents the response function under optical trap with the trap stiffness k. Provided that $A''_k(\omega)$ was obtained in wide frequency range, the full complex response function $A_k(\omega) = A'_k(\omega) - iA''_k(\omega)$ is obtained by using the Kramers-Kronig relation. The response function without optical trap $\alpha(\omega)$ is obtained from the measured $A_k(\omega)$ from the Equation (3) in the main text $\alpha(\omega) = A_k(\omega)/[1 - kA_k(\omega)]$. Finally, generalized Stokes relation $G(\omega) = 1/6\pi a\alpha(\omega)$ gives us the complex shear modulus $G(\omega) = G'(\omega) + iG''(\omega)$ of the surrounding material, where G' and G'' represent elastic and viscous component, respectively. Note that FDT becomes $P(\omega) = 2k_B T\alpha''(\omega)/\omega$ when the trap stiffness k is negligibly small compared to $|A_k(\omega)|$, that usually occurs at high frequencies.

Active microrheology (AMR)

Mechanical properties measured with passive MR are valid for samples in thermodynamic equilibrium where fluctuations of a probe particle are purely thermal. In samples out of thermodynamic

equilibrium (typically inside of living cells), fluctuation-dissipation theorem is violated. The response of a probe particle to the externally applied force $F(t)$ must be therefore directly measured with Active MR. As written in ref. 25 in the main text, 2 lasers are used in AMR; one is for probing the particle position (830 nm) and the other is for driving the particle (1064 nm). Usually, the trap stiffness k of 830 nm laser is set to be much smaller than that of 1064 nm laser. Focus of the driving laser was oscillated and a sinusoidal force $F(t) = \hat{F}(\omega)e^{i\omega t}$ is applied to the probe particle. Displacement of the probe Particle $u(t) = \hat{u}(\omega)e^{i\omega t}$ is observed by QPD that detects the probe laser. The frequency-dependent response function is obtained as $\langle \hat{u}(\omega) \rangle = \alpha(\omega)\hat{F}(\omega)$. G' and G'' are given from the $\alpha(\omega)$ by using generalized Stokes relation, $G(\omega) = 1/6\pi a\alpha(\omega)$.

Note S2. Sample preparations

Escherichia coli cell extracts were prepared as described previously in ref. 42 in the main text. Briefly, *E. coli* cells were collected during the log phase of bacterial growth and washed with double-distilled water to remove extracellular proteins, particularly periplasmic proteins. After a wash, the cells were frozen and then dissolved in the same weight of double-distilled water. The cells were lysed by sonication (S-4000 sonicator, MISONIX, Farmingdale, NY). The suspension was centrifuged at 30,000 × *g* for 1 h, and the supernatant was collected.

The cytoplasm of HeLa cells (CC-01-40-25) was purchased from Cilbiotech (Mons, Belgium). According to the manufacturer's instructions, HeLa cells were harvested during the exponential growth phase at 5×10^6 /mL. After a wash with PBS, the HeLa cells were resuspended and incubated in a hypotonic solution for 15 min. Cell membranes were then broken using a Dounce homogenizer. To inhibit cytoskeleton polymerization, 0.1 mM cytochalasin B was added to the extraction buffer throughout the procedure. The cytoplasm was separated from the nuclei and the lipid fraction by centrifugation for 5 min at 13,000 × *g*. The samples were then frozen and afterwards used after thawing and removing the insoluble aggregates by centrifugation at 1770 × *g* for 30 min at 4°C.

Xenopus eggs were washed with Modified Barth's saline, pH 7.8 (MBS) (*1, 2*). Egg jelly was removed by incubation with MBS containing 2% cysteine. The buffer was then removed, and 50 μg/mL cytochalasin B was added to the eggs. After centrifugation at 800 × *g* for 10 s, the supernatants were removed. The cytoplasm was aspirated by a syringe (18G) after centrifugation at 20,000 × *g* for 10 min. The cytoplasm was further centrifuged at 15,000 rpm for 10 min, and the transparent fraction was collected. The collected cell extract of *Xenopus* eggs was resuspended in a buffer containing the energy mix 1/20 vol. and Ca^{2+} at 800 μM. To inhibit cytoskeleton polymerization, 0.1 mM cytochalasin B was present throughout the sample preparation.

Giant spheroplasts of *E. coli* were prepared as described elsewhere (ref. 43 in the main text). Briefly, cells of *E. coli* strain BL21 Star (DE3) harboring a plasmid encoding the GFP gene under control of the T7 promoter were collected during the exponential growth phase. The harvested *E. coli* was treated with lysozyme to disrupt the cell walls. The lysozyme-treated cells were cultivated in a rich medium containing DNase I and penicillin G for 18 h at 37°C. These conditions inhibit cell division; however, they permit the cells to grow in size.

References in Supplementary

1. Chiba, M., Miyazaki, M. & Ishiwata, S. Quantitative analysis of the lamellarity of giant liposomes prepared by the inverted emulsion method. *Biophys. J.* **107**, 346–354 (2014).

2. Kubota, Y. & Takisawa, H. Determination of initiation of dna-replication before and after nuclear formation in xenopus egg cell-free-extracts. *J. Cell Biol.* **123**, 1321–1331 (1993).

3. Gittes, F., Schnurr, B., Olmsted, P. D., MacKintosh, F. C. & Schmidt, C. F. Microscopic viscoelasticity: shear moduli of soft materials determined from thermal fluctuations. *Phys. Rev. Lett.* **79**, 3286–3289 (1997).

4. Maruyama, K., Kaibara, M. & Fukada, E. Rheology of F-actin. *Biochim. Biophys. Acta* **371**, 20–29 (1974).

5. Hinner, B., Tempel, M., Sackmann, E., Kroy, K. and Frey, E. Entanglement, elasticity, and viscous relaxation of actin solutions. *Phys. Rev. Lett.* **81**, 2614 (1998)

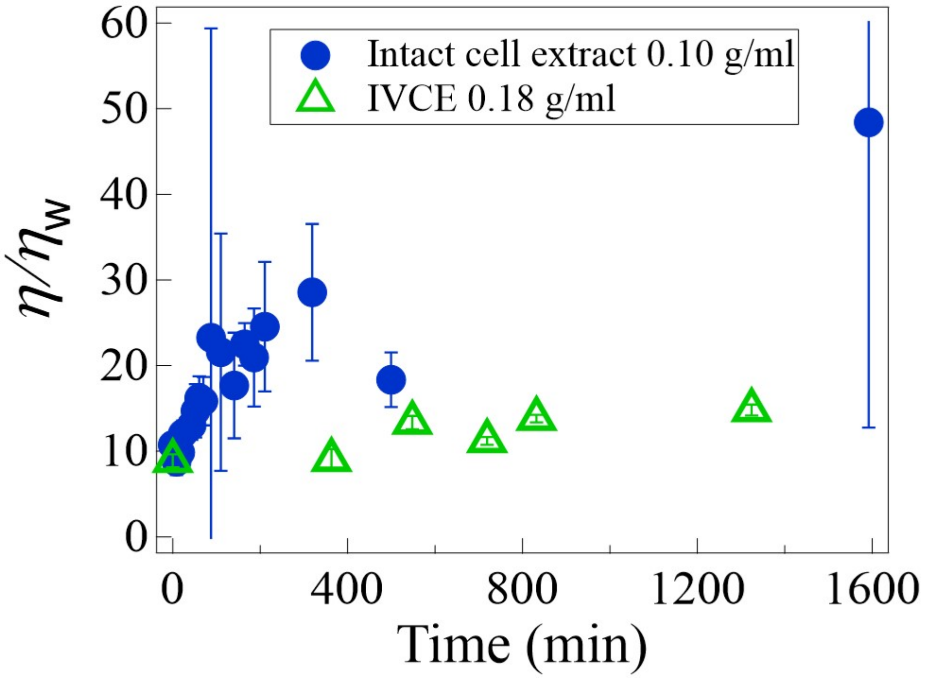

Fig. S1. Aging of the intact cell extract and the metabolite-deficient cell extract

Viscosity η of intact (blue circles) and *in vitro* cell extract that lack metabolism (IVCE: green triangles) of *E. coli* are plotted as a function of time after sample preparation. Each data point is the average measured with PMR using 5 different particles in cell extracts.

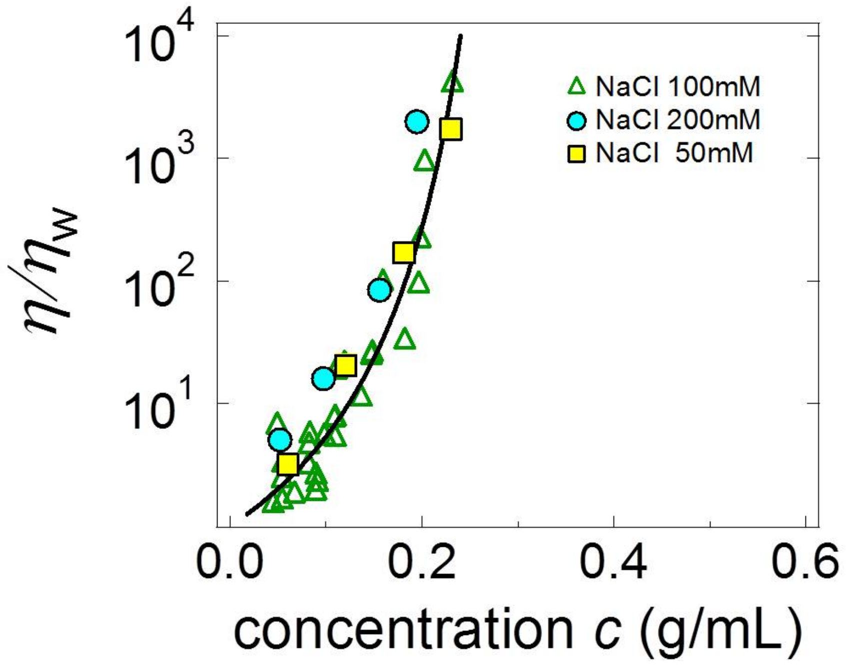

Fig. S2. Effects of salt (NaCl) concentrations on IVCE viscosity

Concentration dependence of viscosity η of IVCEs prepared in buffers containing 50, 100, and 200 mM NaCl (yellow circles, green triangles and light blue circles, respectively) in addition to 20 mM Hepes. η_w is the viscosity of water. In the range of samples measured, ionic strength in buffer does not show significant effects on viscosity.

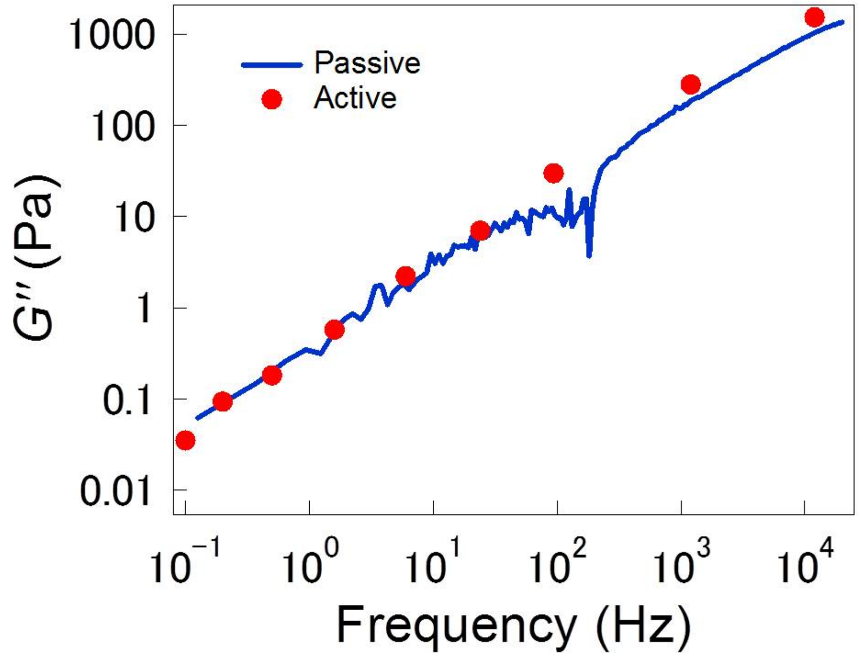

Fig. S3. Shear modulus *G″* measured with active and passive microrheology

Imaginary part of complex shear modulus *G″* of 0.088 g/mL of *E. coli* IVCE plotted as a function of frequency. *G″* obtained with AMR (red cilcles) and PMR (blue line) are shown in red circles and the blue line, respectively. Complete agreement verifies that the IVCE is in thermodynamic equilibrium.

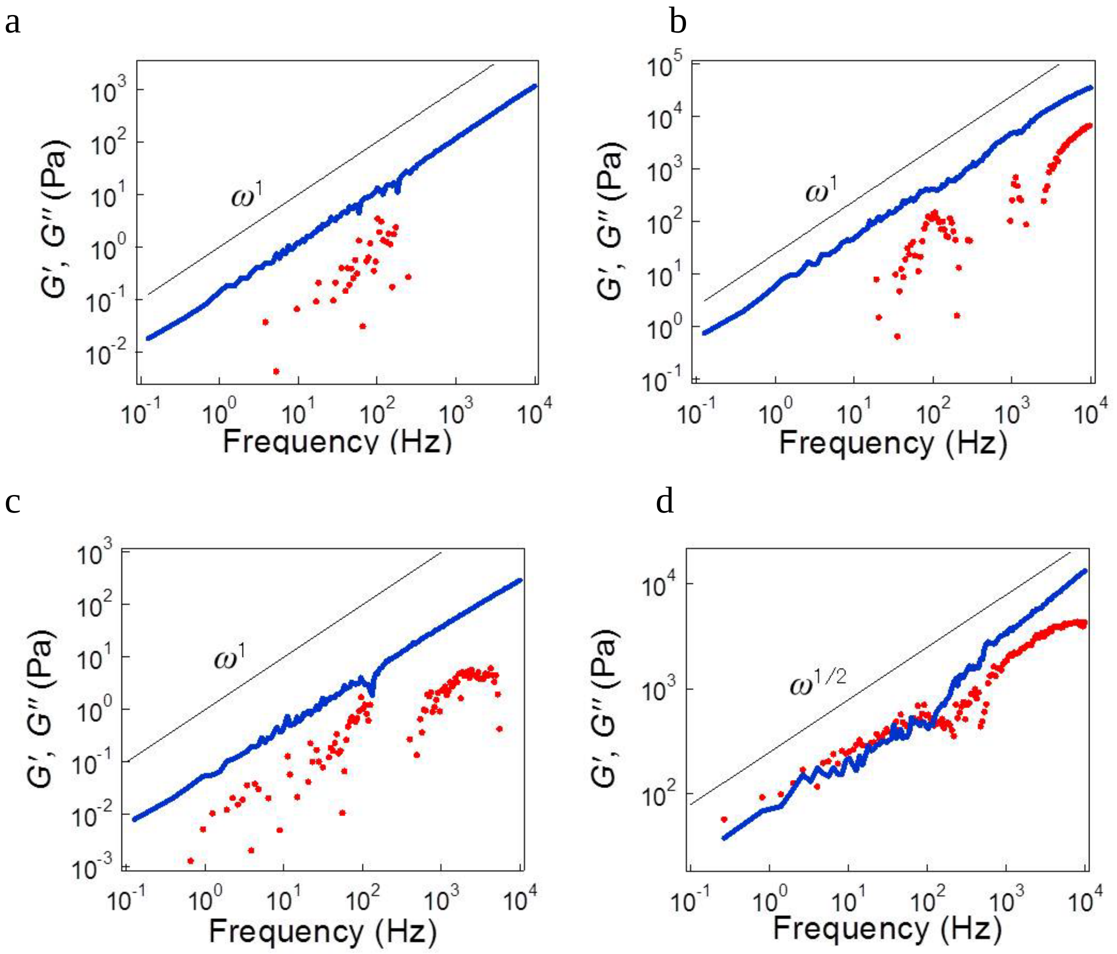

Fig. S4. Fluctuation power spectrum in model cytoplasm measured with passive microrheology

Complex shear moduli G' (red points) and G'' (blue solid line) obtained with PMR are shown as a function of frequency. (a) BSA 0.37 g/mL (b) BSA 0.60 g/mL. (c) IVCE 0.11 g/mL, (d) IVCE 0.23 g/mL. Black solid lines indicate the frequency dependence in the form of power-laws $\propto \omega^{\varepsilon}$. The power-law exponents of G'' deviates from $\varepsilon = 1$ as the macromolecular concentration becomes close to the physiological concentration ~ 0.30 g/mL. This extremely slow relaxation is consistent to the concept of glassy cytoplasm and explains the apparent difference of viscosity measured with active and passive microrheology (see Figure 2d in the main text).

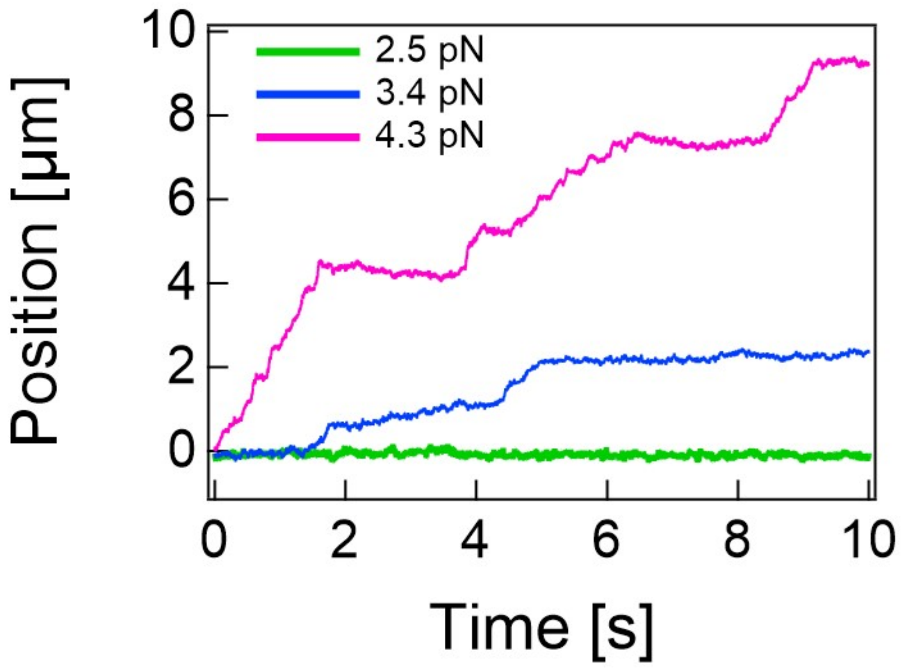

Fig. S5. Bead-pulling MR in weakly-crosslinked actin gels

Trajectories of probes that were embedded in actin gels (1.3 mg/ml) crosslinked with H-meromyosin (0.04 mg/ml) were measured by bead-pulling MR. A probe stayed at the rested position when pulled by a force (2.5 pN: green curve) less than a certain threshold value. Probes moved with intermittent jumps and hops when surrounding matrix yielded with larger force applications (3.4 pN: blue curve and 4.3 pN: pink curve).

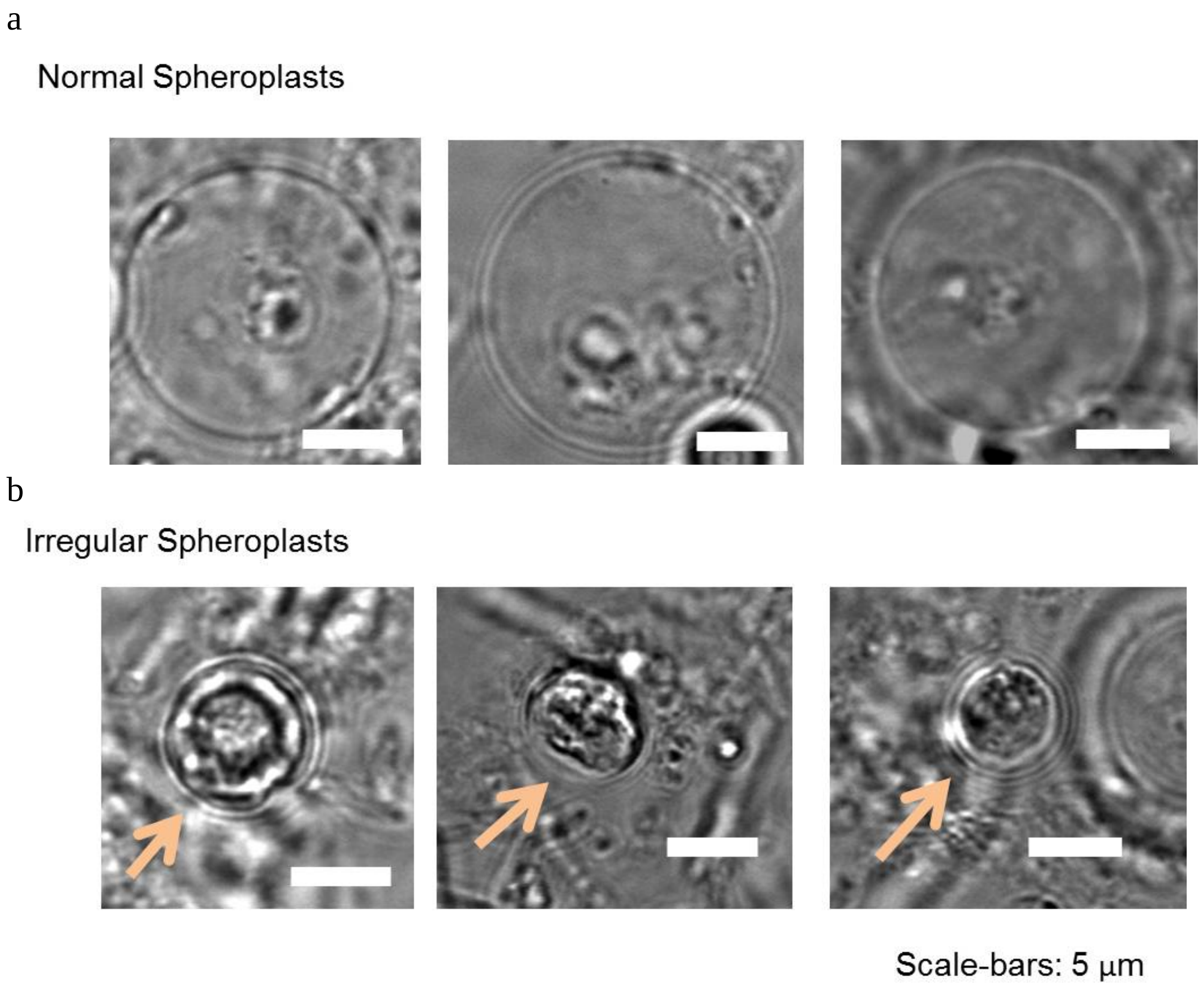

Fig. S6. Microscope images of giant spheroplasts

Blight field images of typical giant spheroplasts (taken 18h after cell-wall disruption). (a) Majority (~ 99%) of spheroplasts show regular spherical shape with optically homogeneous ingredients inside. (b) Small portion (~ 1%) of spheroplasts (indicated by arrows) takes irregular size, shapes and shows inhomogeneous intracellular texture.

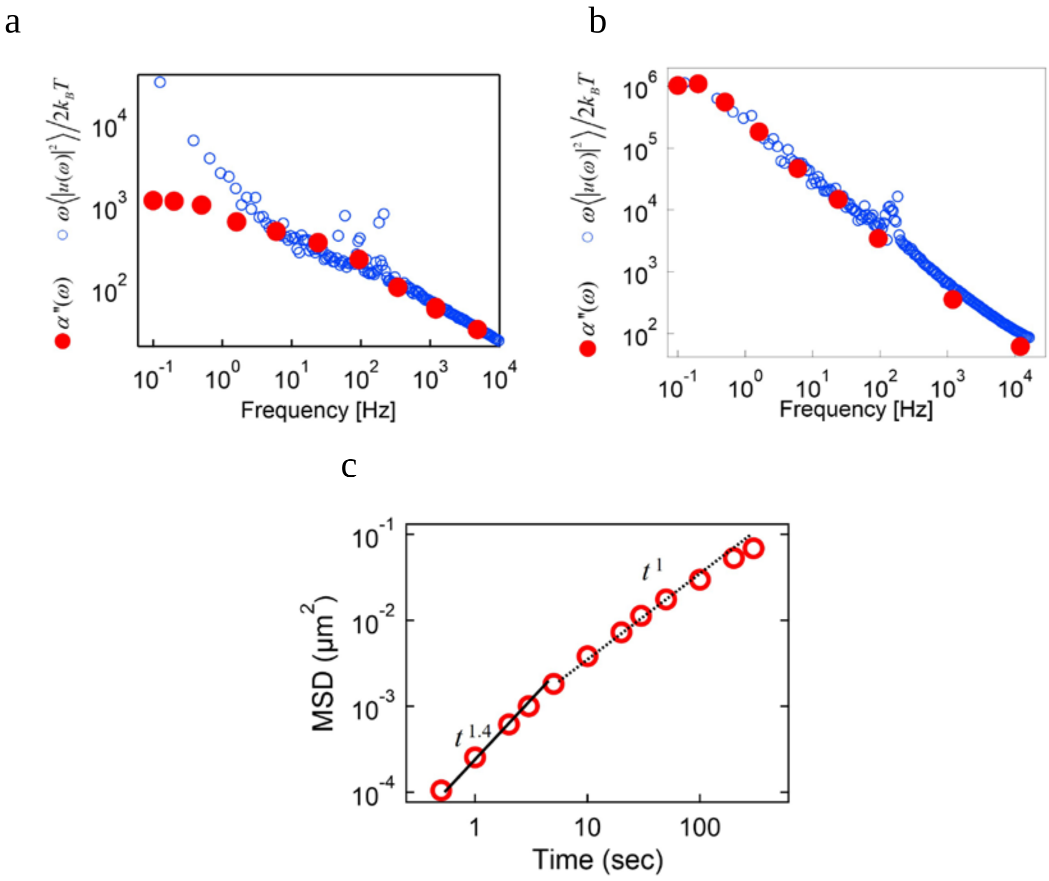

Fig. S7. FDT is violated with the slow ballistic fluctuations in living cells.

Imaginary part of response function $\alpha''(\omega)$ measured by active microrheology (open blue circles) and $\omega \cdot P(\omega)/2k_BT$ measured by passive microrheology (filled red circles) (a) in a living HeLa cell (b) in a cell extract (*E. coli* 0.09 g/ml). In living cells, $\omega \cdot P(\omega)/2k_BT$ shows larger values than $\alpha''(\omega)$ at low frequencies. This indicates that FDT is violated since fluctuation of the probe $\omega \cdot P(\omega)/2k_BT$ is activated by non-thermal forces in cells. At high frequencies, however, $\omega \cdot P(\omega)/2k_BT$ and $\alpha''(\omega)$ agrees, which indicates that FDT is satisfied. (c) Mean-squared displacements $\mathrm{MSD} = \langle (x - x_0)^2 \rangle$ in a living cell (NIH3T3) measured by passive microrheology (open red circles). Power-low exponent of the MSD showed crossover from ballistic ($\propto t^{1.4}$: solid line) regime to slow diffusive ($\propto t^{1}$: broken line) fluctuations.

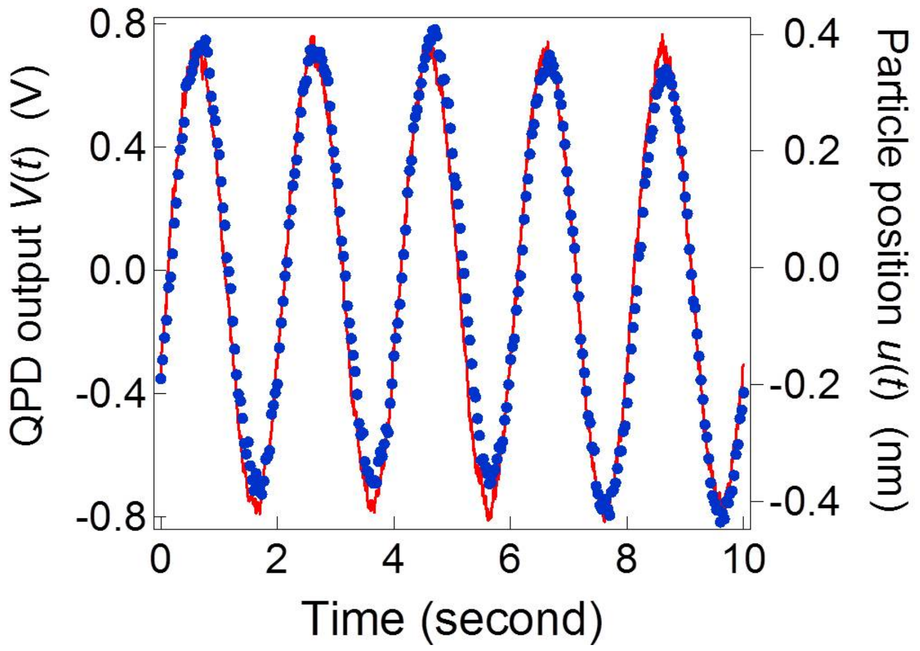

Fig. S8. The direct calibration of QPD output for particle displacements.

A probe particle was optically trapped and sinusoidally oscillated by using the drive laser (λ = 1064 nm). The output voltage $V(t)$ of the QPD that measures the diffraction of the fixed probe laser (λ = 830 nm) is shown as the red curve. Particle positon $u(t)$ was simultaneously measured by a video camera and shown by the blue points. The linear relation between $V(t)$ and $u(t)$ for small displacements gives us the calibration factor $Cal \equiv u(t)/V(t)$. The refractive index of samples used in this study such as highly concentrated BSA solution, cell extracts and also living cells are dependent on solute concentrations and therefore mostly unknown. The QPD output voltage was therefore directly calibrated in this way for each particle in every sample.

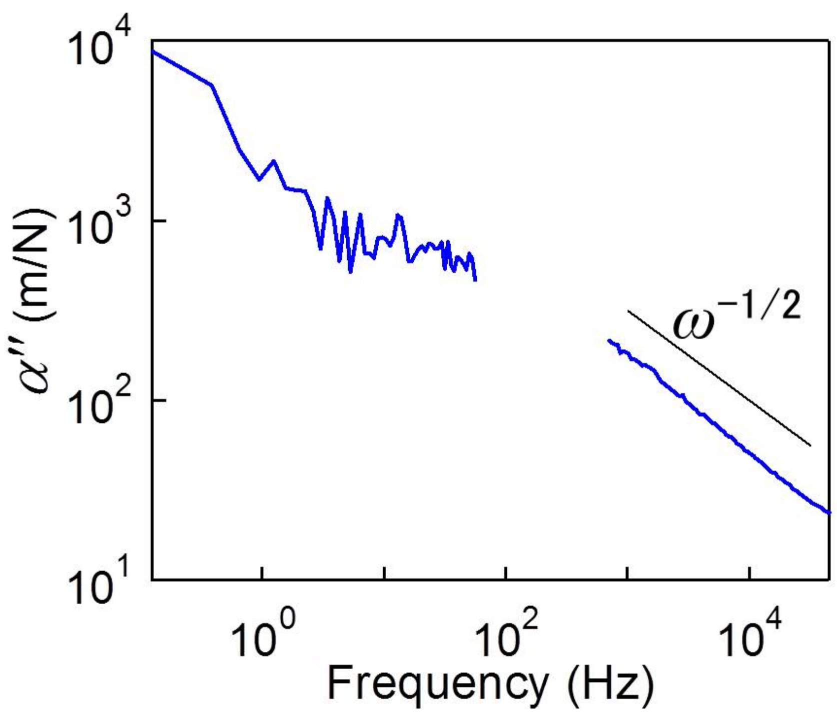

Fig. S9. Probe fluctuation in a living HeLa cell

Imaginary part of response function α'' in a living HeLa cell. Power-low of α'' to high frequency range ($< 10^4$ Hz) show $\alpha \sim (i\omega)^{-0.5}$. Data from 100 [Hz] to 700 [Hz] in this figure are not shown because of electrical and mechanical noises present at corresponding frequencies.

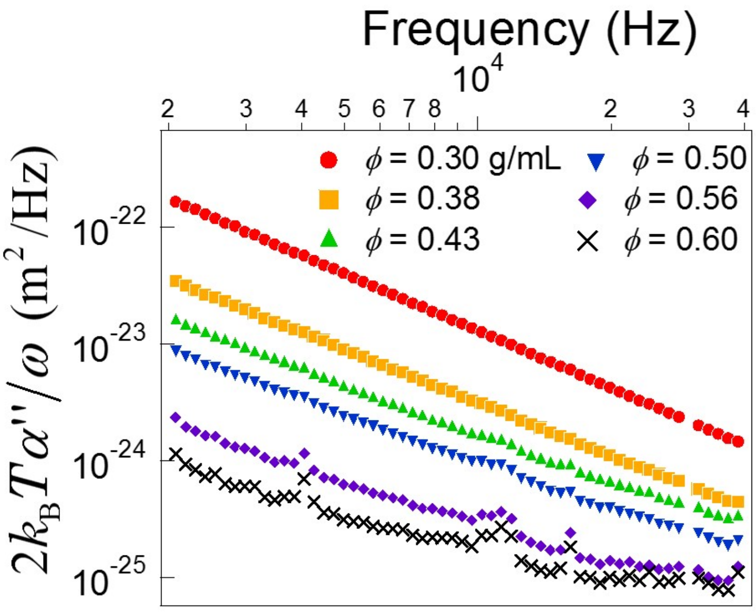

Fig. S10. High-frequency probe fluctuations in living cells (HeLa)

Power spectrum of high-frequency fluctuations $P(\omega)=2k_BT\alpha''/\omega$. The osmolarity of the environment was varied from isotonic (red circles) to hypertonic by adding sucrose to the culture media up to the final concentration of 750 mM (black cross marks).

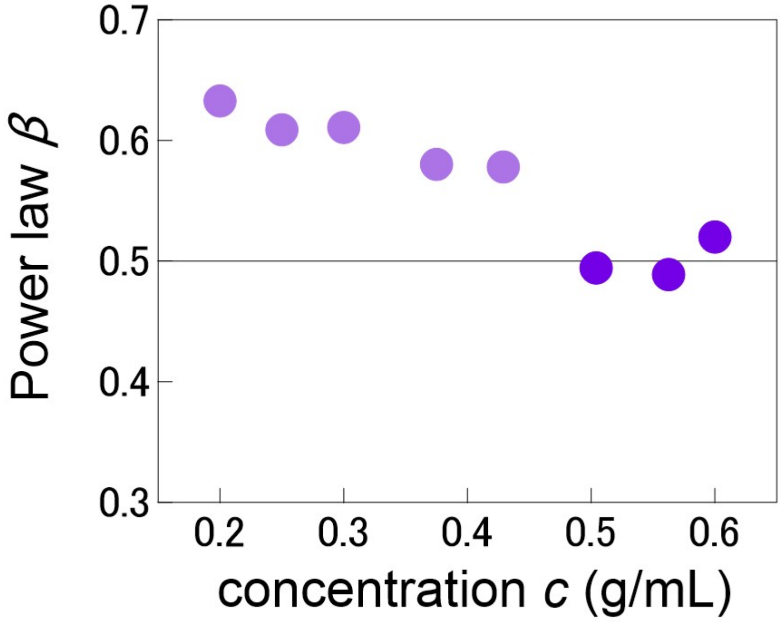

Fig. S11. Exponents of power-law response $\alpha(\omega) \propto \omega^{-\beta}$ in living cells (NIH3T3)

High-frequency response of probes embedded in NIH3T3 cells was fitted with power-law function $\propto \omega^{-\beta}$ and the exponent β was plotted as a function of concentration. As it is written in the main text, β = 1/2 was found for *in vitro* and *living* cytoplasm in the crowded condition (> 0.2 g/mL) (Supplementary Figs. S4d and S6) which we attribute to their glass-forming property. On the other hand, in living NIH3T3 cells, β is distinct from 1/2 at low concentrations (0.2 – 0.43 g/mL). In exactly the same range, η in living NIH3T3 cells deviates from the exponential dependency. It is well-known that semiflexible polymer networks such as cytoskeletons exhibit a larger exponent β = 3/4 (3). The cytoskeletons are poor in the confluent layer of epithelium sheets (MDCK and HeLa) except membrane cortex from which our probe particles are separated. The isolated flattened/adhered fibroblasts (NIH-3T3), on the other hand, are known to express rich actin cytoskeletons all over their bodies. We therefore believe that NIH-3T3 data (0.2 – 0.43 g/mL) are more affected by cytoskeletons; the intermediate exponent $\beta \approx 0.6$ supports this view. Since the cytoskeletal networks increase their viscosities merely in proportion to concentrations (4, 5), the glass-forming property of condensed cytoplasm becomes dominant at higher concentrations (c > 0.5 g/mL); β becomes 1/2 and η shows exponential increase with concentrations (Fig. 5 in the main text).

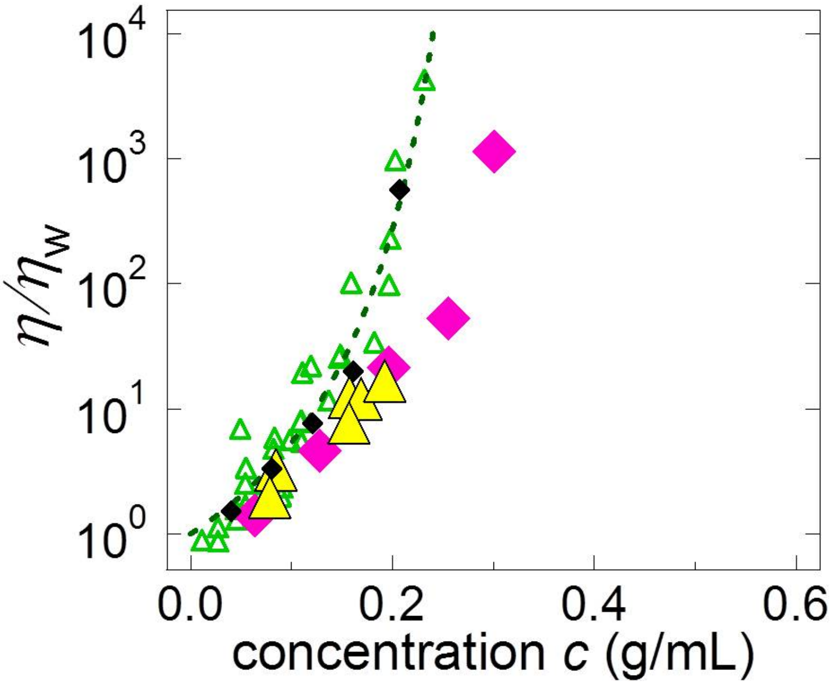

Fig. S12. The effects of centrifugation on viscosity in Cell extract.

Concentration dependence of relative viscosity η/η_w (η_w: viscosity of water) of cell extracts (big yellow triangles: *E. coli* extract prepared by using Lysozyme and freeze-thaw, big pink diamonds: HeLa cells S100 extract, small green triangles: *E. coli*, small black diamonds: HeLa cells). The broken line is the fit of Equation 1 in the main text to cell extracts data. Small green triangles, small black diamonds and the broken line are the same as those in Figure 1 in the main text. Smaller biomacromolecules were selectively collected by the procedures below. When smaller biomacromolecules were selectively collected, e.g., by purification with ultracentrifugation, the viscosity of cell extracts was slightly affected

<u>HeLa cells S100 extract (big pink diamonds)</u> were purchased from CILBIOTECH (CC-01-41-50, Mons, Belgium). According to the manufacturer's instruction, supernatants after another 100,000× g 2h centrifugation of normal Hela cell extracts (CC-01-40-50, small black diamonds) were collected. Larger macromolecules are therefore less in these extracts.

<u>Cell extracts of *E. coli* prepared by using Lysozyme and freeze-thaw (big yellow triangles)</u>: Cell walls and membranes of *E. coli* were partially disrupted by sequential treatments of Lysozyme and freeze-thaw for the extraction of cytoplasm. Since several fractions of large macromolecules still remaining in cells, this gentle extraction collects smaller molecules compared to the extracts prepared by complete disruption of cells with sonication (Figure S9 in the Supporting Information).

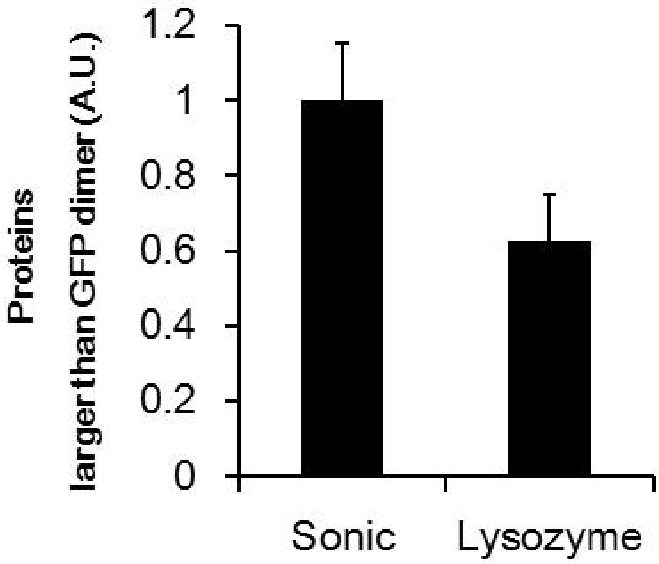

Fig. S13. Amounts of large macromolecules in cell extract prepared by combination of Lysozyme and freeze-thaw are smaller than those by sonication.

Cells at late log-phase were treated with Lysozyme, immersed in double-distilled water, and then disrupted by freeze-thaw. The obtained cell extracts retained efficient activity of cell-free protein expression similar to IVCE in this study. Protein levels in the cell extracts prepared by the Lysozyme treatment and sonication were estimated from the levels of blue intensities derived from coomassie brilliant blue staining analyzed by ImageJ software. Fractions of proteins larger than superfolder GFP dimer in each sample evaluated by native agarose gel electrophoresis (R. Kim *et al.* 2011, CSH protocol) were shown (n = 3, standard deviations were indicated by the error bars).

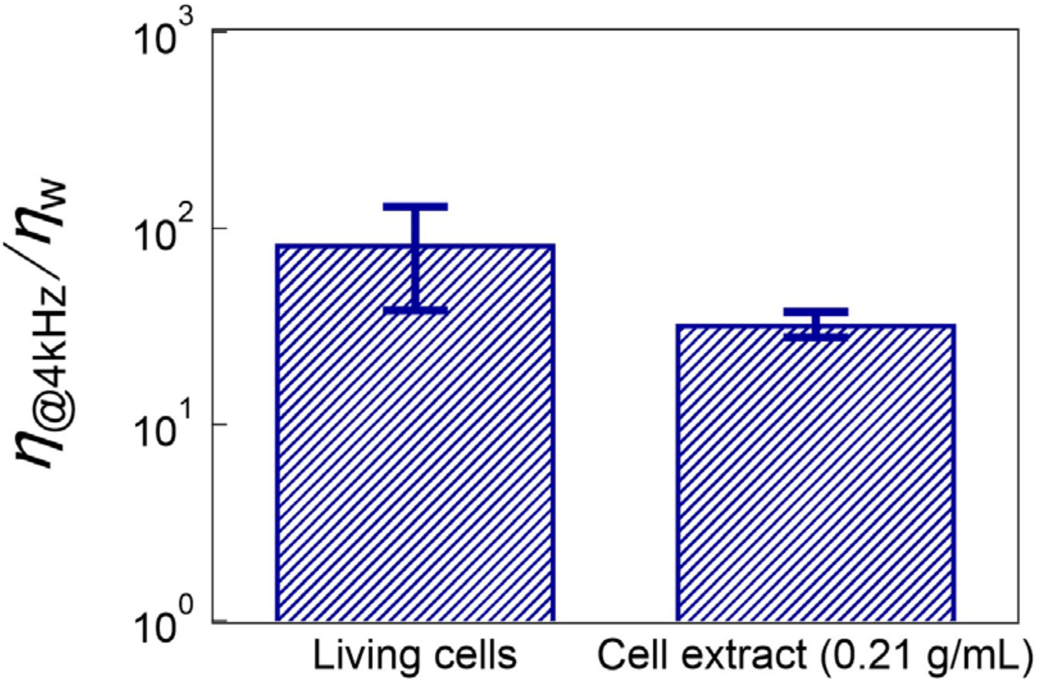

Fig. S14. Distribution of mechanical properties of cell extracts and in living cells

η in living HeLa cells (n = 9) and that of concentrated 0.21 g/ml cell extracts (n = 9) were measured with PMR and their averages and standard deviations are shown.

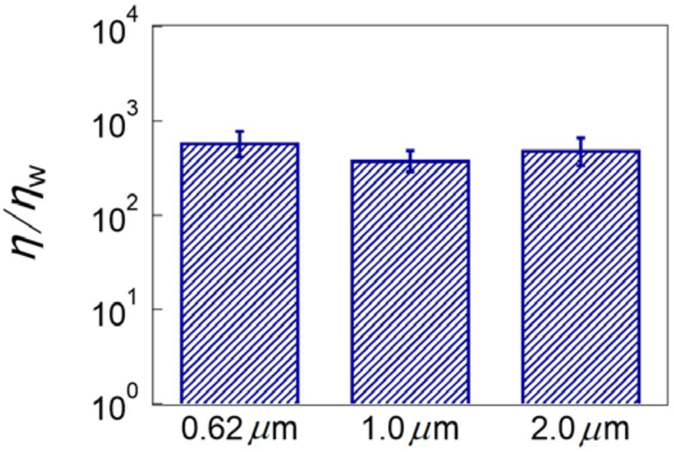

Fig. S15. Probe size dependency in highly concentrated Cell extracts (0.21 g/ml)

η of concentrated cell extracts (0.21 g/ml) were measured with PMR. PEG-coated probes with three different sizes ($2a$ = 0.62 μm, 1 μm and 2 μm) were embedded in the same sample. Averages of η and standard deviations are obtained from 16 particles for each size of probes.

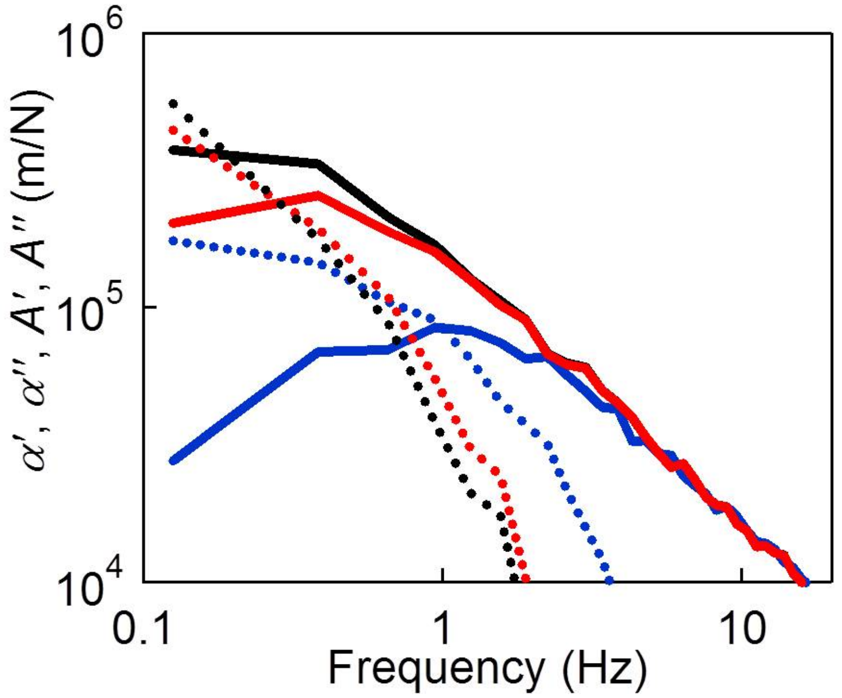

Fig. S16. Probe fluctuation suppressed by trapping forces.

Real (dotted curves) and imaginary (solid curves) parts of complex response functions, A_k (red), A_{3k} (blue) and α (black), measured with PMR in the same BSA solution (0.54 g/mL) as that shown in Figure 6b in the main text. Imaginary parts A''_k, A''_{3k} and α'' were directly obtained from the thermal fluctuation via FDT. Real parts, (A'_k, A'_{3k} and α') were calculated from corresponding imaginary parts via Kramers-Kronig relation.
